# Therapeutic potential of natural arginase modulators: mechanisms, challenges, and future directions

**DOI:** 10.3389/fphar.2025.1514400

**Published:** 2025-04-22

**Authors:** Ting Li, Jieying Wang, Huan Wang, Bowei Zhang, Lijuan Duan

**Affiliations:** ^1^ Department of Neurosurgery, West China Hospital, Sichuan University, Chengdu, China; ^2^ Clinical Trial Center, West China Hospital, Sichuan University, Chengdu, China; ^3^ West China School of Nursing, Sichuan University, Chengdu, China; ^4^ School of Pharmacy, Chengdu University of Traditional Chinese Medicine, Chengdu, China; ^5^ Southwest Institute of Technical Physics, Chengdu, China

**Keywords:** natural arginase modulator, cancer therapy, cardiovascular disease, neuroprotection, macrophage polarization

## Abstract

Arginase (Arg) plays a pivotal role in numerous pathological processes, with its dysregulated expression being intricately associated with tumor progression and immune evasion. This review comprehensively examines the diversity, mechanisms, and clinical potential of natural Arg modulators, encompassing polyphenols, flavonoids, and terpenoids. These bioactive compounds exert their modulatory effects on Arg activity through multiple mechanisms, including direct enzyme interaction, regulation of signaling pathways, and modulation of cellular metabolism. The therapeutic potential of these metabolites spans across various medical domains, notably in cardiovascular diseases, oncology, neurological disorders, and inflammatory conditions. Specifically, polyphenol metabolites such as resveratrol and curcumin have demonstrated significant benefits in cardiovascular health and neuroprotection, while flavonoids including rutin and quercetin have shown promising effects on intracellular inflammatory factors and tumor cell proliferation. Similarly, terpenoids like perillyl alcohol and triptolide have been found to influence cell polarization processes. However, despite their substantial therapeutic potential demonstrated in experimental studies, the development of natural Arg modulators faces several significant challenges. These include complexities in drug design attributed to the intricate structure and multiple isoforms of Arg, difficulties in elucidating precise mechanisms due to Arg’s multifaceted roles in various metabolic pathways, and limitations in current drug delivery systems. To overcome these challenges, future research should focus on continuous optimization of experimental design paradigms, enhancement of experimental models and data quality, thorough evaluation of therapeutic efficacy, and strategic integration of natural Arg modulators with precision medicine approaches.

## Graphical Abstract

**Figure:**
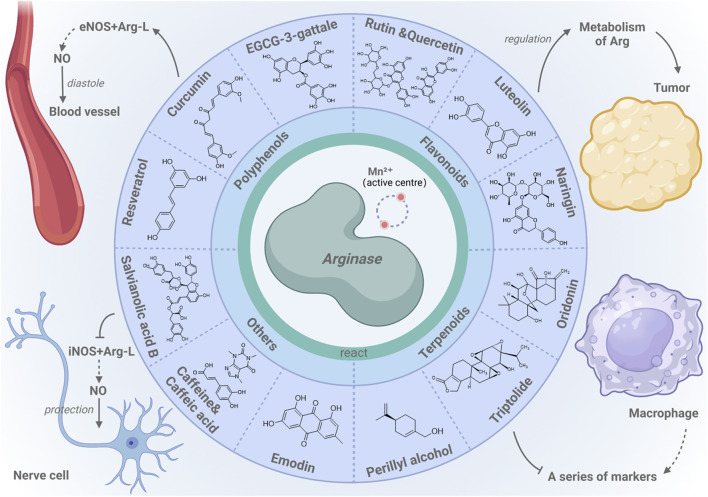
Arginase-centric metabolic modulation and cellular health. This review highlights the regulation of metabolic pathways by arginase and its impact on cell function, highlighting arginase’s interactions with natural modulators such as polyphenols, flavonoids, and terpenes, which are essential for the health of various cell types.

## 1 Introduction

Arg, a pivotal enzyme with diverse roles across multiple tissues, is fundamental to arginine (Arg-L) metabolism. In the final stage of the urea cycle, Arg plays a key role in ammonia detoxification and polyamine (PA) synthesis by splitting Arg-L to form urea and L-ornithine, both of which are essential for cellular function and tissue repair (Pérez and Rius-Pérez, 2022). In addition, Arg is directly or indirectly involved in proline synthesis and fibrosis. In terms of proline synthesis, Arg indirectly influences proline production by regulating Arg-L metabolism (Li et al., 2021; Zhang et al., 2023). Proline, in turn, plays a significant role in cellular stress responses and collagen synthesis, being essential for tissue repair and fibrosis (Han et al., 2021; Liu et al., 2024). During the fibrosis process, the activity of Arg is closely related to collagen deposition and the expression of fibrosis markers. It influences the generation of nitric oxide (NO) and polyamines (PAs) by regulating the availability of Arg-L, thereby modulating fibrosis-related signaling pathways. Moreover, Arg exerts significant regulatory effects on the immune system by modulating the responses of T cells and macrophages to external stimuli. In tumor biology, Arg reduces the material basis of tumor cell growth by consuming Arg-L, significantly affecting tumor proliferation and immune evasion, providing novel insights into tumor cell metabolism regulation.

Given Arg’s complex roles in both physiological and pathological contexts, researchers are actively exploring strategies to effectively modulate its activity. Arg modulators have attracted considerable attention due to their potential therapeutic benefits and favorable safety profiles. Recent studies have highlighted the potential of Arg modulators in addressing intractable medical problems. For example, cancer treatment still faces many challenges. As reported by the NIH in 2022 (Bray et al., 2024), approximately 9.7 million people die from cancer, with colorectal cancer accounting for 9.3 percent of cancer deaths and breast cancer accounting for 6.9 percent of cancer deaths. Studies have shown that Arg modulators can indirectly affect Arg-L metabolism by regulating the concentration of inflammatory factors such as interleukin. This, in turn, modulates the urea cycle and PA synthesis pathways, ultimately influencing cell proliferation and apoptosis, thereby improving cancer-related outcomes. In addition, in response to the 2023 WHO report that 1.28 billion adults aged 30–79 years suffer from hypertension (World Health Organization, 2023), Arg modulators play a key role in physiological processes such as vasodilation and inflammatory response by regulating Arg-L metabolism and NO production. This provides new avenues for the treatment of hypertension and related cardiovascular conditions.

In this review, we provide a comprehensive overview of the biological activities, mechanisms of action, and potential applications of natural Arg modulators in disease treatment. Our analysis used key words such as “natural metabolites,” “arginase” and “regulation” to conduct a preliminary screening of the article. Subsequently, we further screened natural metabolites with therapeutic effects for specific diseases such as “hypertension,” “cancer,” “macrophage inflammatory,” “neurodegenerative diseases” and other keywords. In this review, we conducted a comprehensive review of current research and clinical trials using PubMed, Google Scholar, and SciFinder databases. All selected papers were published in peer-reviewed scientific journals. Experimental exclusion criteria include: effective dose is less than 1 g/kg/d, experimental model and method criteria are evaluated in strict accordance with the principles of “based on experiment, determination mechanism, pharmacological evidence, visual data.” In addition, all the regulatory effects of traditional drugs introduced in this paper are supported by scientific tests. By systematically examining the existing literature, we elucidate the current status and future trends in this field. Our analysis not only furnishes researchers with precise information and profound insights but also underscores the unique advantages and potential of natural Arg modulators. We anticipate that this review will foster deeper understanding and stimulate further exploration of this critical area, inspiring future research directions.

## 2 Classification and sources of arginase modulators: from chemical synthesis to natural origins

### 2.1 Structural characteristics of arginase

**FIGURE 1:**
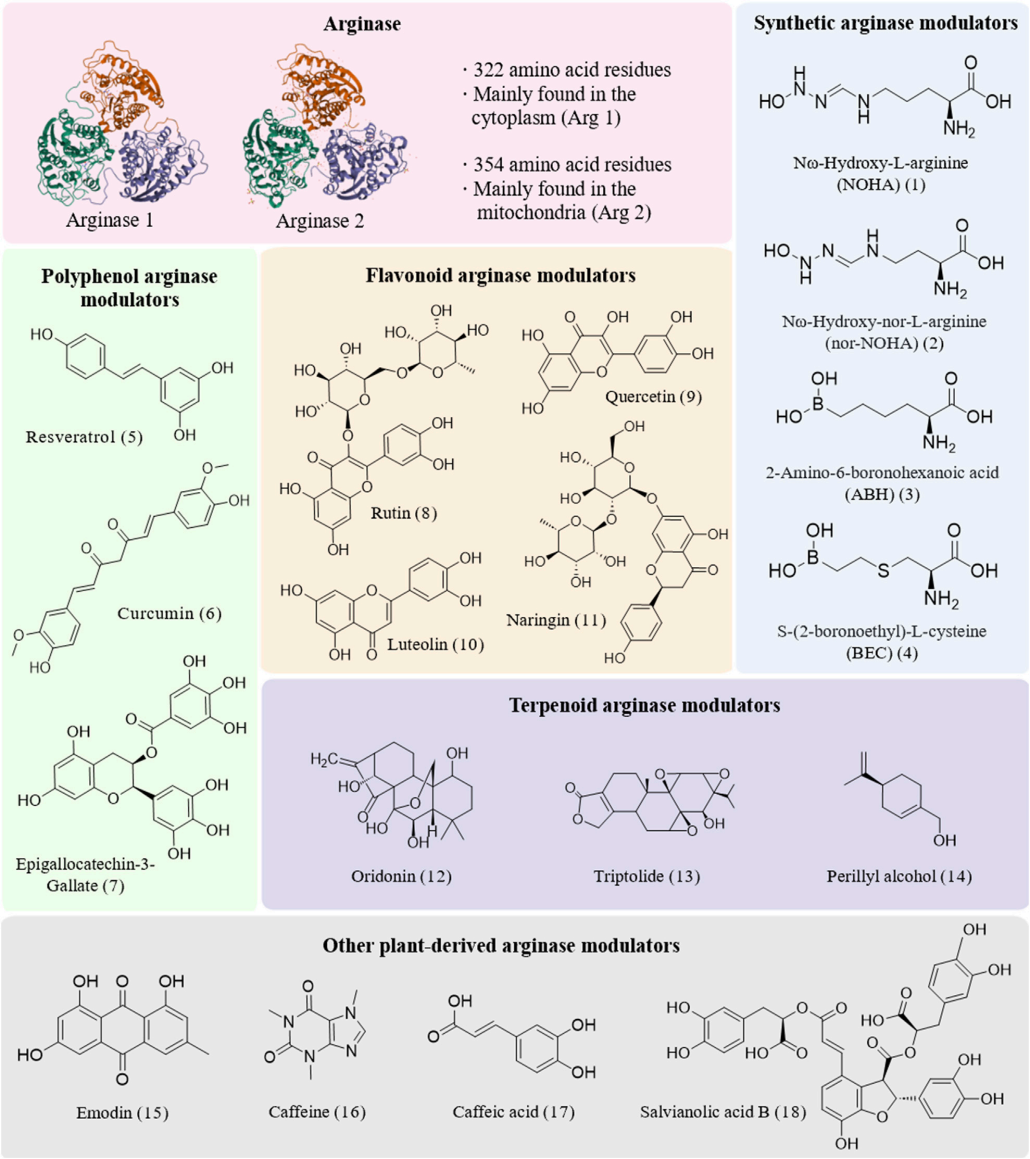
Structures of arginase and diverse modulators.

Arg is a group of metalloenzymes that play pivotal roles in biological systems (Niu et al., 2022). Among these, the two isoforms (Figure 1), arginase 1 (Arg1) and arginase 2 (Arg2), each fulfill distinct biological functions. Arg 1, comprising 322 amino acid residues, is predominantly located in the cytoplasm and is highly expressed in the liver. Its coding gene resides on chromosome 6q23. In contrast, Arg 2 consists of 354 amino acid residues and is distributed across the kidney, prostate, small intestine, and lactating mammary gland, primarily functioning within mitochondria. The gene encoding Arg 2 is positioned on chromosome 14q24. The catalytic centers of these metalloenzymes contain two essential metal ions, typically manganese, that are essential for enzymatic activity. These catalytic sites enhance the precision of Arg binding to the substrate. Such structural insights not only clarify the precise catalysis by Arg but also hold significant potential for the development of small-molecule and natural modulators to modulate their activity, thereby impacting therapeutic strategies for specific disease conditions.

### 2.2 Relationship between arginase and disease

With its distinctive molecular structure, Arg serves as a pivotal enzyme in regulating Arg-L metabolism and NO production, profoundly influencing the pathogenesis of numerous diseases. Deficient Arg activity (Therrell et al., 2017; Scaglia and Lee, 2006; Schlune et al., 2015; Crombez and Cederbaum, 2005; Wiechert et al., 1989) can lead to hyperargininemia, characterized by progressive spastic paralysis (Tariq et al., 2017; McNutt et al., 2023; Sin et al., 2015), cognitive and motor dysfunction (Lin et al., 2023), seizures (Pavuluri et al., 2023), and growth and developmental abnormalities (McNutt et al., 2023). Conversely, excessive Arg activity impedes the synthesis of the vasodilator NO by inhibiting the interaction between Arg-L and endothelial nitric oxide synthase (eNOS). This can result in endothelial dysfunction (Gambardella et al., 2020) and aberrant blood pressure regulation, as observed in conditions such as pre-eclampsia (Haworth et al., 2021), erectile dysfunction (ED) (Lacchini et al., 2015), and myocardial ischemia/reperfusion (MI/R) injury (Tratsiakovich et al., 2013). In neurological disorders, Arg mitigates neurodegenerative changes (Moretto et al., 2019) induced by inflammation by curbing the overproduction of pro-inflammatory NO. In oncology, Arg suppresses tumor progression by depleting Arg-L, thereby obstructing its metabolic support for tumor cells. Additionally, as a biomarker of immune cell polarization (Rath et al., 2014), Arg offers novel insights into macrophages states and paves the way for innovative therapeutic strategies in treating immune-related diseases like asthma (Boonpiyathad et al., 2019) and intestinal inflammation (Huang et al., 2022). These multifaceted roles of Arg underscore its significance as a potential therapeutic target and provide a scientific foundation for developing novel therapeutic strategies across various diseases, heralding a potential transformation in clinical treatment paradigms.

### 2.3 Research progress and challenges of synthetic arginase modulators

In terms of modulating Arg activity, chemically synthesized Arg modulators (Pudlo et al., 2017; Abdelkawy et al., 2017) have emerged as pivotal agents in the realm of Arg modulation. Their design strategies predominantly focused on emulating the substrate structure of Arg, leading to the development of diverse metabolites (Figure 1). Notably, α-amino acid derivatives, particularly Nω-hydroxy-L-arginine (NOHA) **(1)** (Bollenbach et al., 2019) and Nω-hydroxy-nor-L-arginine (nor-NOHA) **(2)** (Chauhan et al., 2024), are the first metabolites to exhibit micromolar modulatory effects by replacing the hydroxyl bridging ion of Arg with n-hydroxyl and have been widely used in laboratory settings. Furthermore, boronic acid derivatives, such as 2-amino-6-boronohexanoic acid (ABH) **(3)** (Golebiowski et al., 2013) and S-(2-boronoethyl)-L-cysteine (BEC) **(4)** (Kim et al., 2001), mimic transition-state analogs by forming hydrogen or ionic bonds with active sites, thereby playing crucial roles in laboratory research. Inspired by the significant effects of these modulators, researchers have endeavored to incorporate boronic acid groups into the side chains of α-amino acids and explore the bonding of various metal-binding groups, such as sulfonamides, imidazoles, and nitro groups, to the binuclear manganese clusters in Arg. This approach has demonstrated broad applicability in laboratory investigations. Additionally, reaction coordinate analogs—including amino acid aldehydes (Shin et al., 2004) and epoxides—as well as certain existing drugs like chloroquine (Bak et al., 2023) and antiretroviral agents (Lisi et al., 2014), have shown substantial utility in experimental studies due to their Arg modulatory properties. These strategies not only offer novel avenues for treating related diseases but also provide fresh perspectives for drug design and repurposing.

While chemically synthesized Arg modulators have demonstrated significant modulatory effects in laboratory settings, their transition to clinical applications is hindered by several challenges. These challenges are primarily related to pharmacokinetic properties (Abdelkawy et al., 2017), particularly concerning bioavailability and stability. For instance, ABH and certain derivatives exhibit oral bioavailability of less than 5%, coupled with a brief half-life *in vivo* of approximately 15–30 min. This results in rapid metabolism of these chemical metabolites within the body, causing a swift decline in effective concentrations, and making it difficult to sustain prolonged efficacy. Additionally, safety concerns—notably the toxic effects of boronic acid derivatives on cellular physiological functions—represent significant hurdles in the development of chemical modulators. These potential toxicity issues necessitate thorough evaluation and resolution through comprehensive toxicological studies. Consequently, for these synthetic Arg modulators to be viable in clinical settings, researchers must further refine the metabolite structures and enhance their pharmacokinetic profiles to ensure drug safety.

Advancing strategies to regulate Arg activity requires addressing the limitations of synthetic modulators by exploring the potential of natural counterparts. Synthetic and natural modulators exhibit profound multidimensional distinctions in origin, design, activity, mechanism, and pharmacokinetic properties. Synthetic modulators are the product of deliberate chemical engineering; for instance, ABH, a mimic of the Arg-L transition state, chelates manganese ions within Arg’s active site, while pyrrolidine analogs employ rigid cyclic structures to restrict conformational flexibility and enhance binding affinity (Pham et al., 2018). Conversely, natural modulators, primarily sourced from plant extracts, include polyphenols and flavonoids such as chlorogenic acid and pterostilbene, highlighting their intrinsic biological origins. In terms of activity, synthetic modulators offer superior potency and precision, with compounds such as OATD-02 displaying an IC_50_ of 20 nM against Arg-1 (Molaro et al., 2025)—markedly outperforming chlorogenic acid’s IC_50_ of 10.6 μM (Yacout et al., 2014). However, their mechanisms are often unidimensional, typically incapable of modulating Arg activity through multifaceted pathways. In contrast, natural modulators exhibit more intricate regulatory mechanisms, indirectly influencing Arg activity via antioxidant properties or nitric oxide (NO) pathway modulation. Pharmacokinetically, synthetic modulators frequently encounter challenges, including low bioavailability—evident in early boronic acid derivatives requiring prodrug optimization—and chemical instability, which may elevate toxicity risks. Natural modulators, due to their biological derivation, demonstrate higher safety profiles, faster metabolic elimination, and reduced toxicity. For example, CAPA’s activity decreases tenfold in humans (Pham et al., 2016), underscoring its inherent safety advantages in clinical applications. Nonetheless, synthetic modulators such as Numidargistat have progressed to clinical trials in solid tumors, whereas natural modulators largely remain confined to *in vitro* investigations.Looking forward, the integration of precision synthetic design with the structural and functional diversity of natural modulators holds promise for developing efficient, low-toxicity Arg-regulation strategies. The complementary strengths of these modulators could unlock novel therapeutic avenues for complex pathological conditions, thereby addressing unmet clinical needs with enhanced safety and efficacy.

### 2.4 Research and application of natural arginase modulators

In contrast to synthetic modulators, natural Arg modulators (Girard-Thernier et al., 2015) exhibit significant potential for development and application due to their naturally complex structures (Figure 1) and superior biocompatibility. These natural metabolites—including polyphenols, flavonoids, and terpenoids—demonstrate both direct and indirect mechanisms in modulating Arg activity, offering promising therapeutic avenues for cardiovascular diseases, cancer (Chen et al., 2021), cell polarization and neurodegenerative disorders. Additionally, other natural metabolites such as emodin, caffeine, and salvianolic acid B have been identified to exert substantial regulatory effects on Arg activity, partially filling the gap in the treatment of inflammatory diseases.

Pharmacological studies have further elucidated the multipath regulatory effects and double-sided regulation of natural Arg modulators across various diseases. In cardiovascular conditions, these modulators effectively mitigate inflammation-induced vascular endothelial dysfunction (Caldwell et al., 2010; Johnson et al., 2010; Johnson et al., 2013; Krause et al., 2015; Bhatta et al., 2017), such as hypertension, by reducing Arg activity and its competition with eNOS, thereby enhancing vasodilator NO synthesis. On the contrary, in neurological disorders (Lange et al., 2004; Eren et al., 2024), Arg modulators enhance the competition with inducible nitric oxide synthase (iNOS) by increasing Arg activity, thus preventing excessive synthesis of pro-inflammatory NO and ultimately combating inflammation-induced neurodegeneration. In oncology, Arg modulators impede tumor cell proliferation and metastasis by inhibiting Arg activity and reducing PA synthesis. Furthermore, by modulating immune cell polarization (Rath et al., 2014), Arg activity inhibition can avert inappropriate immune responses, offering new therapeutic opportunities for respiratory diseases such as asthma.

We will further elaborate on the functional performance of these natural metabolites in experimental studies and their potential clinical applications. Through comprehensive analysis of the biological activities and mechanisms of these natural Arg modulators, we aim to provide a robust scientific foundation for future drug development and clinical applications.

## 3 Mechanisms of natural arginase modulators

### 3.1 Polyphenols: selective effect on competitive reaction between arginase and nitric oxide synthase

**FIGURE 2:**
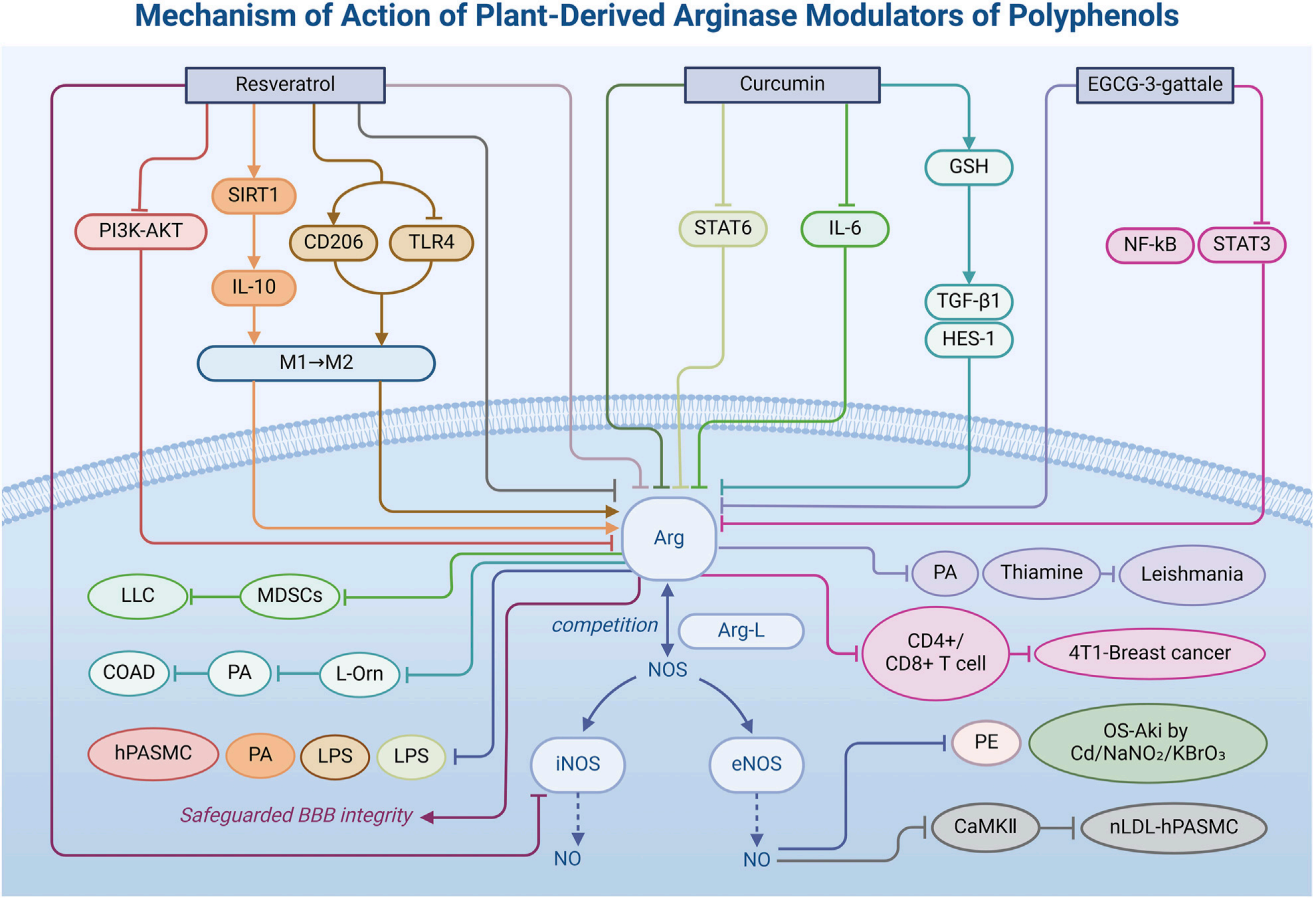
Mechanism of action of natural arginase modulators of polyphenols.

Polyphenolic metabolites, a diverse group of plant secondary metabolites, are renowned not only for their antioxidant properties but also for their multifaceted potential in modulating Arg activity within the biomedical domain. They play a role in maintaining the normal basic function of cells, primarily through changing the competitive binding force of Arg in the metabolic reaction of Arg-L, thus regulating the synthesis amount of NO. This section delves into specific polyphenolic metabolites, elucidating their mechanisms (Figure 2)—both direct and indirect—in regulating Arg activity (Table 1) and highlighting their prospective applications in the treatment of inflammatory diseases, mainly cardiovascular diseases.

**TABLE 1:** Effects of natural arginase modulators.

Category	Metabolites	Plant origin	Disease	Model	Dose/Duration	Result	Pathway	Effect on Arg activity	Effects on biomarkers	Ref.
Polyphenol	Resveratrol	*Veratrum album* L	PE	Plasma from pregnant women with PE	Processing of umbilical artery samples: 100 µM for 4 h	Improved the blood pressure in pregnant women	Combined Arg active sites	↓	NO ↑	Bueno-Pereira et al. (2022)
	Resveratrol	*Veratrum album* L	Pulmonary hypertension	hPASMC under hypoxic conditions	40, 80 and 100 µM for 0.5, 24, 48 and 120 h	Inhibited the proliferation of hPASMCs under hypoxic conditions	Inhibited the PI3K-Akt pathway	↓	Phospho-Akt ↓ Expression of Arg mRNA ↓	Chen et al. (2014)
	Resveratrol	*Veratrum album* L	Hyperlipemia	nLDL induced RASMCs	/	Reversed the RASMCs proliferation induced by native nLDL	Downregulated the CaMKII expression	↓	NO ↑ CaMKII ↓ RASMCs ↓	Trieu Dieu Linh et al. (2016)
	Resveratrol	*Veratrum album* L	RA	CFA induced AIA in male Wistar rats	50 mg/kg for 30 d	Reduced the edema of the rat posterior paw	Activated SIRT1 pathway to promote M2 cells polarization	↑	IL-10 ↑	Wang et al. (2022)
	Resveratrol	*Veratrum album* L	LPS	LPS-induced M1 polarization of RAW264.7 macrophages	2, 4 and 8 µM for 24 h	Reduced the leakage of LPS	Promoted the transformation of M1 macrophages into M2 macrophages	↑	CD206 mRNA ↑ Protein and mRNA expression levels associated with the TLR4 signaling pathway ↓	Fan et al. (2021)
	Resveratrol	*Veratrum album* L	EAE	EAE mouse	10, 25 and 50 mg/kg for 20 d	Alleviated the paralysis symptoms and EB leakage caused by EAE	Inhibited the disease-induced overexpression of the proinflammatory transcripts iNOS and IL-1β	↑	iNOS ↓ IL-1β ↓ Inflammatory mediator ↓	Wang et al. (2016)
	Curcumin	*Curcuma longa* L	Heavy metal induced nephropathy	Renal toxicity induced by cadmium	12.5, 25 mg/kg for 7 d	Restored kidney metabolism, blood flow, and function	Inhibited the effects from cadmium exposure on renal ADA and Arg activities	↓	ADA ↓ NO ↑ Control: 63.1 ± 6.1 mg/dL; Cd: 91.2 ± 4.1 mg/dL; Cd + curcumin 12.5 mg/kg: 70.1 ± 8.1 mg/dL; Cd + curcumin 25 mg/kg: 69.1 ± 6.9 mg/dL	(Akinyemi et al., 2017)
	Curcumin	*Curcuma longa* L	Heavy metal induced nephropathy	Renal toxicity induced by NaNO_2_	20 mg/kg for 28 d	Reduced the oxidative stress kidney injury induced by NaNO_2_	Regulated ADA and Arg activity	↓	ADA ↓ NO ↑ Control: 6.76 ± 1.65 mg/dL; NaNO_2_: 13.93 ± 2.45 mg/dL; Curcumin: 8.93 ± 1.22 mg/dL; Curcumin + NaNO_2_: 7.67 ± 1.17 mg/dL	Adewale et al. (2021)
	Curcumin	*Curcuma longa* L	Heavy metal induced nephropathy	Renal toxicity induced by KBrO_3_	20 mg/kg for 7 d	Reduced the oxidative stress kidney injury induced by KBrO_3_	Decreased Arg activity and increased NO level	↓	Creatinine ↓ Blood urea ↓ Electrolyte ↓ Control: 6.76 ± 1.65 mg/dL; KBrO_3_: 17.17 ± 2.85 mg/dL; Curcumin: 8.93 ± 1.22 mg/dL; KBrO_3_ + curcumin: 7.67 ± 0.95 mg/dL	Akomolafe et al. (2021)
	Curcumin	*Curcuma longa* L	Antineoplastic drug-induced cognitive impairment	Cyclophosphamide induced cognitive model in rats	20 mg/kg for 14 d	Improved the cognitive dysfunction in rats	Alleviated the stimulating effect of cyclophosphamide on Arg	↓	NO ↑ Protein and non-protein thiols ↑ MDA ↓ caspase-3 ↓	Akomolafe et al. (2020)
	Curcumin	*Curcuma longa* L	WAT	WAT mouse	0.4% (w/w) for 14 weeks	Reduced the degree of WAT adiposity and total macrophage infiltration	Inhibited Stat6 mechanisms to reduce WAT fat and total macrophage infiltration in mice	↓	LPS ↓ Stat6 ↓	Islam et al. (2021)
	Curcumin	*Curcuma longa* L	lung cancer	Lewis lung cancer syngeneic tumor model	50 mg/kg for 24 h	Decreased the tumor size of lung cancer	Inhibited the function of IL-6 producing cells	↓	IL-6 ↓	Liu et al. (2016)
	Curcumin	*Curcuma longa* L	Colon cancer	DMH-induced colon cancer model	60 mg/kg/day for 14 d	Inhibited the colon cancer tumor growth	Restored the expression level of GSH and upregulated the expression of TGF-β1 and HES-1 mRNA	↓	L-ornithine ↓ PA (spermine and spermidine) ↓	(Bounaama et al., 2012)
	EGCG-3-gallate	*Camellia sinensis* (L.) Kuntze	Leishmaniasis	Leishmania Arg and rat liver Arg model	10, 100, 1,000 µM for 15 min IC_50_ = 3.8 μM	Inhibited the reproductive differentiation of Leishmania	Formed hydrogen bonds to active sites of Arg and interacted with enzyme-substrate complexes	↓	PA ↓ Thiamine ↓	Goncalves dos Reis et al. (2013) Carter et al. (2021)
	EGCG-3-gallate	*Camellia sinensis* (L.) Kuntze	breast cancer	breast cancer cell	250, 500, 1,000 and 2000 μg/mL for 30 d	Decreased the immunosuppressive ability of breast cancer cells	Intervened NF-κB and STAT3 signaling pathways	↓	CD4^+^/CD8^+^ T cell ↑	Xu et al. (2020)
Flavonoid	Rutin and its aglycone quercetin	*Ruta graveolens* L	ED	Paroxetine induced ED rat model	25, 50 mg/kg for 14 d	Improved the relaxation function of corpus cavernosum smooth muscle	Inhibited Arg activity to increase the production of vasodilator	↓	NO ↑ PDE-5 ↓ AChE ↓ ACE ↓	Adefegha et al. (2018)
	Rutin and its aglycone quercetin	*Ruta graveolens* L	Neuroglioma	Co-culture model of glioma cells (C6 cells) and microglia	In the proliferation assay: 50 µM for 24 h In the migration experiment: 50 µM for 24 and 48 h	Impaired the proliferation and migration of tumor cells	Inhibited Arg mRNA	↓	NO ↑	da Silva et al. (2020)
	Rutin and its aglycone quercetin	*Ruta graveolens* L	Neurodegenerative diseases	BV-2 cell	12.5, 25 and 50 μg/mL for 1 h	Protected neuronal cells and improve neurodegenerative diseases	Promoted the transformation of microglia from pro-inflammatory M1 phenotype to anti-inflammatory M2 phenotype to enhance the M2 marker Arg activity	↑	TNF-α ↓ IL-1β ↓ IL-6 ↓ NO ↓ CD206 ↑ IL-10 ↑	Lang et al. (2021)
	Luteolin	*Reseda odorata* L	Leishmaniasis	Parasite cells	L-arginine used: 12.5, 25, 50 and 100 mM Luteolin used ranging from 1.25 to 30 μM	Increased oxidative stress in parasite cells, achieving the effect of infection control	Inhibited Arg activity by forming Mn^2+^ -Mn^2+^ metal bridge between luteolin and Arg active site	↓	PA synthesis ↓	(Manjolin et al., 2013)
	Luteolin (luteolin-7-diglucoside and luteolin-7-glucoside)	*Reseda odorata* L	Vasodilatation	An isolated aortic ring model of L-NAME on acetylcholine-induced vasodilation	100 µM for 30 min IC_50_ = 95.3 ± 9.5 μM	Reversed the inhibitory effect of NO synthase inhibitor L-NAME on acetylcholine-induced vasodilation	Inhibited Arg activity to enhance eNOS activity	↓	NO ↑	Arraki et al. (2020)
	Luteolin (Luteolin-7-O-glucoside LUT7G)	*Reseda odorata* L	Gastric ulcer	Experimental model of ethanol-induced ulcer	25 mg/kg for 4 d	Attenuated the damage of NO to gastric mucosal cells	Activated Arg to promote the conversion of Arg-L to PA	↑	NO ↓ PGE2 ↑ HSP-70 ↑	Antonisamy et al. (2016)
	Naringin	*Citrus maxima* (Burm.) Merr.	Hippocampal damage	A rat model of hippocampal injury induced by hypertension and purine metabolism defects	50 mg/kg for 14 d	Improved hippocampal damage	Inhibited Arg activity, NO/cGMP and cAMP/PKA signaling pathways	↓	NO/cGMP ↓ cAMP/PKA ↓	Akintunde et al. (2022)
	Naringin	*Citrus maxima* (Burm.) Merr.	ED	Mouse model of ED induced by exposure to L-NAME (an NOS inhibitor) and the BPA	80 mg/kg for 14 d	Improved ED	Inhibited Arg activity Activated NOS/cGMP/PKG signaling pathway	↓	ACE ↓ PDE-5 ↓ AChE ↓	Akintunde et al. (2020a)
	Naringin	*Citrus maxima* (Burm.) Merr.	Cataracts	Mouse model of cataract induced by exposure to L-NAME, an NOS inhibitor, and the BPA	80 mg/kg for 14 d	Improved Cataracts	Reduced free radical damage to cells through antioxidant properties and its ability to stabilize the lipid bilayer of the cell membrane	↓	NO ↑	Akintunde et al. (2020b)
	Naringin	*Citrus maxima* (Burm.) Merr.	I/R injury	I/R injury model of small intestine and lung	50 mg/kg	Promoted tissue recovery after I/R injury	Inhibited Arg activity to increase intensity of eNOS immunoreactivity in the liver and lung	↓	Free radicals ↓ Tissue degradation effect ↓ Oxidative stress response ↓	Cerkezkayabekir et al. (2017)
	Naringin	*Citrus maxima* (Burm.) Merr.	Hyperammonemia	NH_4_Cl-induced hyperammonemia rat model	80 mg/kg	Alleviated the neurotoxicity of ammonia to the brain	Promoted the hydrolysis of Arg-L urea in which Arg is involved	↑	Urea ↑ Blood ammonia ↓	Arumugam and Natesan (2017)
	Naringin	*Citrus maxima* (Burm.) Merr.	Inflammation in M2 microglial cells	M2 microglial cells	5, 10 and 20 µM for 24 h	Promoted the activation of M2-type phenotype microglia	Promoted the activation of M2-type microglia through JAK/STAT3 signaling pathway	↑	IL-1β ↓ IL-6 ↓ TNF-α ↓	Li et al. (2022b)
	Naringin	*Citrus maxima* (Burm.) Merr.	OXL-induced neuropathy	OXL-induced peripheral neuropathy model	50 and 100 mg/kg on Days 1, 2, 5 and 6	Improved the nerve blood flow and nerve conduction function	Reversed OXL-induced oxidative stress and improved intracellular Arg expression	↑	NO ↓	Semis et al. (2022)
Terpenoids	Oridonin	*Isodon rubescens* (Hemsl.) H. Hara	MI/R injury	MI/R model	10 mg/kg for 7 h	Reduced the size of cardiac infarction	Inhibited Arg activity Regulated glycolysis, branched-chain amino acid metabolism, tryptophan and kynurenine metabolism, and bile acid metabolism	↓	NO ↑ Oxidative stress in cardiomyocytes ↓	Zhang et al. (2019)
	Triptolide	*Tripterygium wilfordii* Hook. f	Polarization of M2 macrophages	RAW264.7 macrophage model	20 ng/mL for 24 h IC_50_ = 25.7 nM	Impaired the immunosuppressive function of M2 macrophages	Downregulated IL-4 and IL-10 to inhibit Arg and other markers which can affect the polarization response of macrophages	↓	IL-4 ↓ IL-10 ↓	Li et al. (2020)
	Triptolide	*Tripterygium wilfordii* Hook. f	Arthritis	MDSCs in PBMCs of RA patients AIA mouse model	RA: 1, 5 and 10 nM for 2 d ALA mouse: 0.1 μg/g/d	Weakened the ability of MDSCs to induce Th17 cell polarization and slowed disease progression	Inhibited the differentiation of MDSCs, and reduced Arg protein expression in a dose-dependent manner	↓	MDSCs ↓ IL-17 ↓	(Zhao et al., 2024)
	Triptolide	*Tripterygium wilfordii* Hook. f	CIRI	CIRI induced microglial cells	50 ng/mL for 1 h	Inhibited the apoptosis in HT-22	Downregulated the expression of iNOS and upregulated the expression of Arg to promote the polarization of microglia to M2 type with anti-inflammatory and repair functions Inhibited the activation of CTSS/Fractalkine/CX3CR1 signaling pathway to reduce the M1 polarization of microglia	↑	iNOS ↓ CTSS/Fractalkine/CX3CR1 ↓	(Zhao et al., 2024)
	Perilla alcohol	*Perilla frutescens* (L.) Britton	Parodontopathy	RAW 264.7 mouse macrophages under periodontal disease conditions	10, 25, 50, 100 and 250 µM for 24, 48 and 72 h	Improved the inflammatory response of periodontal disease	Inhibited the expression of Arg induced by IL-4 and regulated STAT6 signaling pathway	↓	IL-4 ↓	Alves Figueiredo et al. (2020)
Others	Emodin	*Rheum palmatum* L	Asthma	DRA induced asthma in mouse	20 mg/kg/day on Days 12, 13 and 14	Alleviated airway inflammation	Reduced the infiltration of eosinophils and lymphocytes in bronchoalveolar lavage fluid, mucus secretion and serum IgE production *In vitro* experiments: inhibited the polarization of AAMs induced by IL-4, STAT6 phosphorylation and KLF4 expression in a dose-dependent manner	↓	Ym-1 ↓ Fizz-1 ↓	Song et al. (2018)
	Emodin	*Rheum palmatum* L	LPS -induced ALI	LPS -induced ALI model	20 mg/kg/day 40 mg/kg/day 80 mg/kg/day	Improved liver damage	Increased the expression of M2 macrophage markers Arg and CD206 in the liver of mice Inhibited the TLR4 signaling pathway to reduce the expression of pro-inflammatory factors TNF-α and IL-6	↑	CD206 ↑ TNF-α ↓ IL-6 ↓ ALT ↓ AST ↓	Ding et al. (2018)
	Emodin (Piceatannol-3-O-β-D-glucopyranoside)	*Rheum palmatum* L	Hypercholesterolemia	A high-cholesterol diet-induced model of vascular disease	5, 10, 25, 50 and 100 μM for 48 h	Reduced fatty streak formation induced by a high cholesterol diet	Inhibited Arg activity to activate NO production	↓	NO ↑	Kim and Ma (2019)
	Caffeine and caffeic acid	*Coffea arabica* L	Memory and cognitive impairment	The rat brain and cerebral cortex model	50 mg/kg Caffeine or caffeic acid for 7 d	Maintained the function of the brain and cerebral cortex	Improved brain function via improvements in the antioxidant status and inhibition of AChE, ADA, and Arg activities	↓	NO ↑	Oboh et al. (2021)
	Caffeine and caffeic acid	*Coffea arabica* L	Hypertension	L-NAME-induced hypertensive rat model	Caffeine (5 mg/kg) Caffeic acid (5 mg/kg or 25 mg/kg) Caffeine (5 mg/kg) + Caffeic acid (5 mg/kg) Caffeine (5 mg/kg) + Caffeine acid (25 mg/kg)	Regulated brain function and improve cognitive health	Reduced the activities of Arg and ACE	↓	NOx ↑ MDA ↓	Akomolafe (2017)
	Caffeic acid	*Coffea arabica* L	Hypertension	Cyclosporin-induced hypertensive rats	10 and 15 mg/kg for 7 d	Reduced hypertension	Reduced the activities of Arg and MDA	↓	NO ↑ MDA ↓ CAT ↑ GSH ↑	Agunloye et al. (2019)
	Caffeic acid	*Coffea arabica* L	Cognitive dysfunction and anxiety-like behavior induced by a high-fat diet	HFD and chronic stress-induced Wistar rat models	50 mg/kg/d for 8 weeks	Promoted BDNF expression and improved cognitive function	Reduced oxidative stress markers, increased antioxidant enzyme activity and decreased inflammatory markers Activated the Wnt/β-catenin pathway and inhibited the activity of GSK-3β	↑	MDA ↓ NO ↓ GSH ↑ SOD ↑ GST ↑ IL-1β ↓ IL-2 ↓ TNF-α ↓ IFN-γ ↓ GSK-3β ↓ BDNF ↑	El-Sayed et al. (2024)
	Caffeic acid	*Coffea arabica* L	Leishmaniasis	Model of co-culture of plasmids containing leishmania Arg gene and Arg	IC_50_ for Leishmania *in vitro*: Proflagellate: 60.8 ± 11 µM, Intracellular amastigote: 21.9 ± 5.0 mM CC_50_ for macrophages in cell experiments:1,221 ± 28 µM	Reduced parasite growth and infectivity	Inhibited Arg activity to increase the production of NO in infected macrophages, which can prompt NO to help host cells kill parasites	↓	NO ↑	Garcia et al. (2019)
	Salvianolic acid B	*Salvia miltiorrhiza* Bunge	Tumor induction by LPS	RAW 264.7 macrophage model	100, 200 and 400 µM for 12 h	Reduced the inflammatory polarization of macrophages induced by LPS	Inhibited LPS-induced TNF-α production	/	TNF-α ↓ HO-1 ↑	(Joe et al., 2012)
	Salvianolic acid B	*Salvia miltiorrhiza* Bunge	Hypertension	Mouse models of liver, kidney, and vascular tissue	50 mg/kg IC_50_ = 1.44 mg/L on human liver microsomes	Dilated the blood vessels	Inhibited Arg activity and promoted NO production	↓	NO ↑	Abdelkawy et al. (2017)

#### 3.1.1 Resveratrol

Resveratrol **(5)**, a natural polyphenolic metabolite derived from the leaves of Japanese knotweed and grapevines, is gaining considerable attention as a potential Arg inhibitor in vascular diseases (Chudzinska et al., 2021). In studies on preeclampsia (Bueno-Pereira et al., 2022), researchers observed that resveratrol treatment effectively doubled plasma Arg-L levels in patients and significantly reduced plasma Arg activity to about half of pre-treatment levels. This enhancement was attributed to resveratrol’s ability to inhibit Arg (EC 3.5.3.1), improve the binding of Arg-L to eNOS (EC 1.14.13.39), and promote the production of the vasodilator—NO. Consequently, this mechanism significantly alleviates hypertension in pregnant women. Furthermore, resveratrol influences enzymatic interconversions and signal transduction pathways. Specifically, in pulmonary hypertension (Chen et al., 2014), resveratrol inhibits Arg transcription through suppression of the phosphatidylinositol 3-kinase-protein kinase B signal pathway (PI3K-Akt) signaling pathway, curbing the hypoxia-induced proliferation of human pulmonary artery smooth muscle cells (hPASMCs). Besides, reduced Arg activity enhances Arg-L’s interaction with eNOS, restoring NO levels and decreasing Ca^2+^/calmodulin-dependent protein kinase II (CaMKII) expression (Choi et al., 2019), which counteracts natural low-density lipoprotein (nLDL)-induced Rat smooth muscle cells (RASMCs) proliferation (Trieu Dieu Linh et al., 2016).

Importantly, resveratrol modulates Arg expression during inflammatory cell polarization (Djaldetti, 2024). As an NAD-dependent deacetylase sirtuin-1 (SIRT1) agonist (Wang et al., 2022), resveratrol boosts anti-inflammatory cytokine interleukin-10 (IL-10) production and promotes alternatively activated macrophage (M2) polarization by restoring SIRT1 pathways in rheumatoid arthritis (RA), thereby elevating Arg activity. In bacterial lipopolysaccharide (LPS)-induced classic activated macrophages (M1) polarization of RAW264.7 cells (Fan et al., 2021), resveratrol significantly increased Arg and CD206 mRNA expression while downregulating protein and mRNA levels linked to the toll-like receptor 4 (TLR4) pathway, facilitating the shift from M1 to M2 macrophages. Notably, in experimental autoimmune encephalomyelitis (EAE) models (Wang et al., 2016), doses of 25 and 50 mg/kg of resveratrol suppressed the overexpression of pro-inflammatory transcripts like iNOS and interleukin-1β (IL-1β) and significantly increased Arg expression in the brain. This regulatory action reduces inflammatory mediator production, attenuates local inflammation, diminishes paralysis symptoms, and prevents Evans blue (EB) leakage caused by EAE, thereby safeguarding blood-brain barrier (BBB) integrity. These findings underscore resveratrol’s potential to modulate Arg activity directly and offer novel therapeutic insights into inflammation and vascular health.

#### 3.1.2 Curcumin

Curcumin **(6)**, a polyphenolic metabolite derived from the rhizomes of *Curcuma longa* L., possesses promising therapeutic potential as an Arg inhibitor in medicine and has garnered considerable interest within the food industry due to its significant renal protective effect (Trujillo et al., 2013). Notably, curcumin exhibits inhibitory effects on cadmium-exposed renal adenosine deaminase (ADA) levels and Arg activity (Akinyemi et al., 2017), which can inhibit the binding process of Arg and Arg-L, and reduce the amount of urea production in the urea cycle (Wu and Morris, 1998; Morris, 2002). Therefore, changes in urea production (control: 63.1 ± 6.1 mg/dL; Cd: 91.2 ± 4.1 mg/dL; Cd + curcumin 12.5 mg/kg: 70.1 ± 8.1 mg/dL; Cd + curcumin 25 mg/kg: 69.1 ± 6.9 mg/dL) were used as evidence of the regulatory effect of curcumin on Arg. Curcumin’s inhibitory effect on Arg enhances the collective antioxidant status and NO levels, reduces cadmium accumulation, and alleviates kidney injury. Additionally, curcumin mitigates oxidative stress-induced kidney injury caused by NaNO_2_ by regulating ADA and Arg activities (Adewale et al., 2021). Its effectiveness is also demonstrated through changes in urea production (control: 6.76 ± 1.65 mg/dL; NaNO_2_: 13.93 ± 2.45 mg/dL; curcumin: 8.93 ± 1.22 mg/dL; curcumin + NaNO_2_: 7.67 ± 1.17 mg/dL). In a KBrO_3_-induced kidney injury model (Akomolafe et al., 2021), curcumin protects renal function by decreasing Arg activity and increasing NO levels, ameliorating oxidative stress, and reducing creatinine, blood urea, and electrolyte levels. Similarly, changes in urea production were used to verify curcumin’s ability to regulate Arg (control: 6.76 ± 1.65 mg/dL; KBrO_3_: 17.17 ± 2.85 mg/dL; curcumin: 8.93 ± 1.22 mg/dL; KBrO_3_ + curcumin: 7.67 ± 0.95 mg/dL). These researches lay a scientific foundation for further exploration of curcumin’s clinical applications in treating nephrotoxicity. Furthermore, curcumin mitigates the cyclophosphamide-induced increase in Arg activity (Akomolafe et al., 2020), demonstrating neuroprotective effects validated through cognitive function tests and enzyme assays.

Beyond its renal protective effect, curcumin demonstrates diverse capabilities in regulating biological pathways. Studies on white adipose tissue (WAT) inflammation (Islam et al., 2021) reveal that curcumin intake significantly reduces WAT adiposity and total macrophage infiltration in mice. This effect is attributed to curcumin’s capacity to maintain intestinal barrier integrity by modifying gut microbiota composition, subsequently reducing LPS leakage, inhibiting the signal transducer and activator of transcription 6 (STAT6) pathway, and suppressing Arg expression, which was specifically manifested as a decrease (about 1.5 times) in the WAT protein level of mice in the HFC group by immunohistochemical staining. In the Lewis lung carcinoma syngeneic tumor model (Liu et al., 2016), curcumin significantly lowers interleukin-6 (IL-6) levels in tumor tissues and serum by modulating IL-6-producing cells. This action reduces downstream signaling activation and decreases Arg transcription and expression in myeloid-derived suppressor cells (MDSCs) (Veglia et al., 2021; Wu et al., 2022), ultimately diminishing their immunosuppressive capacity and contributing to an antitumor effect. In 1,2-dimethylhydrazine (DMH)-induced colon cancer experiments (Bounaama et al., 2012), curcumin attenuates Arg activity by restoring glutathione (GSH) levels and upregulating the mRNA levels of tumor-suppressive proteins such as transforming growth factor-β1 (TGF-β1) and Hes Family BHLH Transcription Factor 1 (HES-1). Compared with the DMH group, Arg activity was reduced by approximately 73%, thereby inhibiting arginine-mediated hydrolysis. This effect leads to decreased levels of L-ornithine and reduced synthesis of PAs such as spermine and spermidine, which mediate tumor cell proliferation, thereby arresting tumor growth.

#### 3.1.3 Epigallocatechin-3-gallate

Epigallocatechin-3-gallate (EGCG-3-gallate) **(7)** (Landis-Piwowar et al., 2013), a catechin derived from green tea, exerts its effects not only on Arg but also on other constitutive proteins. Matheus et al. elucidated a dual mechanism of action wherein EGCG-3-gallate acts as a mixed-type inhibitor (Goncalves dos Reis et al., 2013). It effectively impedes the reproductive differentiation of Leishmania by forming hydrogen bonds with Arg active sites or by interacting with the enzyme-substrate complex, thereby reducing Arg activity (IC_50_ = 3.8 μM). This indicates that EGCG has a strong inhibitory capacity. In subsequent studies by Nicola et al., the inhibition process of EGCG was further elucidated through *in vitro* experiments and molecular docking studies (Carter et al., 2021). The researchers pointed out that EGCG reduces the production of ornithine by inhibiting Arg, thereby affecting the synthesis of PAs and thiamine. This, in turn, impairs the parasite’s ability to survive and infect. In breast cancer cells (Xu et al., 2020), EGCG-3-gallate demonstrates a more adaptable regulatory mode by decreasing Arg mRNA levels and protein activity through interference with nuclear factor kappa B (NF-κB) and signal transducer and activator of transcription 3 (STAT3) signaling pathways. This regulation diminishes Arg expression, reduces intracellular Arg-L consumption, enhances cellular Arg-L availability, increases the CD4^+^/CD8^+^ T-cell ratio (Yang et al., 2023), weakens the immunosuppressive capacity of tumor cells, and enhances immune-mediated tumor attack. These mechanisms illustrate that EGCG-3-gallate not only modulates the intracellular environment by directly inhibiting Arg activity but also impacts immune responses and tumor growth by influencing cellular signaling pathways, highlighting its potential in cancer therapy.

Polyphenolic natural Arg modulators, owing to their exceptional vascular protective and anti-inflammatory effects (Minozzo et al., 2018), hold promise as specialized treatments for vascular diseases. However, clinical studies are sparse regarding the appropriate dosage of these modulators, and the dose-response relationship remains unclear. Consequently, future research must concentrate on defining safe clinical dosage ranges to optimize precision medicine.

### 3.2 Flavonoids: regulatory effect on inflammatory factors

**FIGURE 3:**
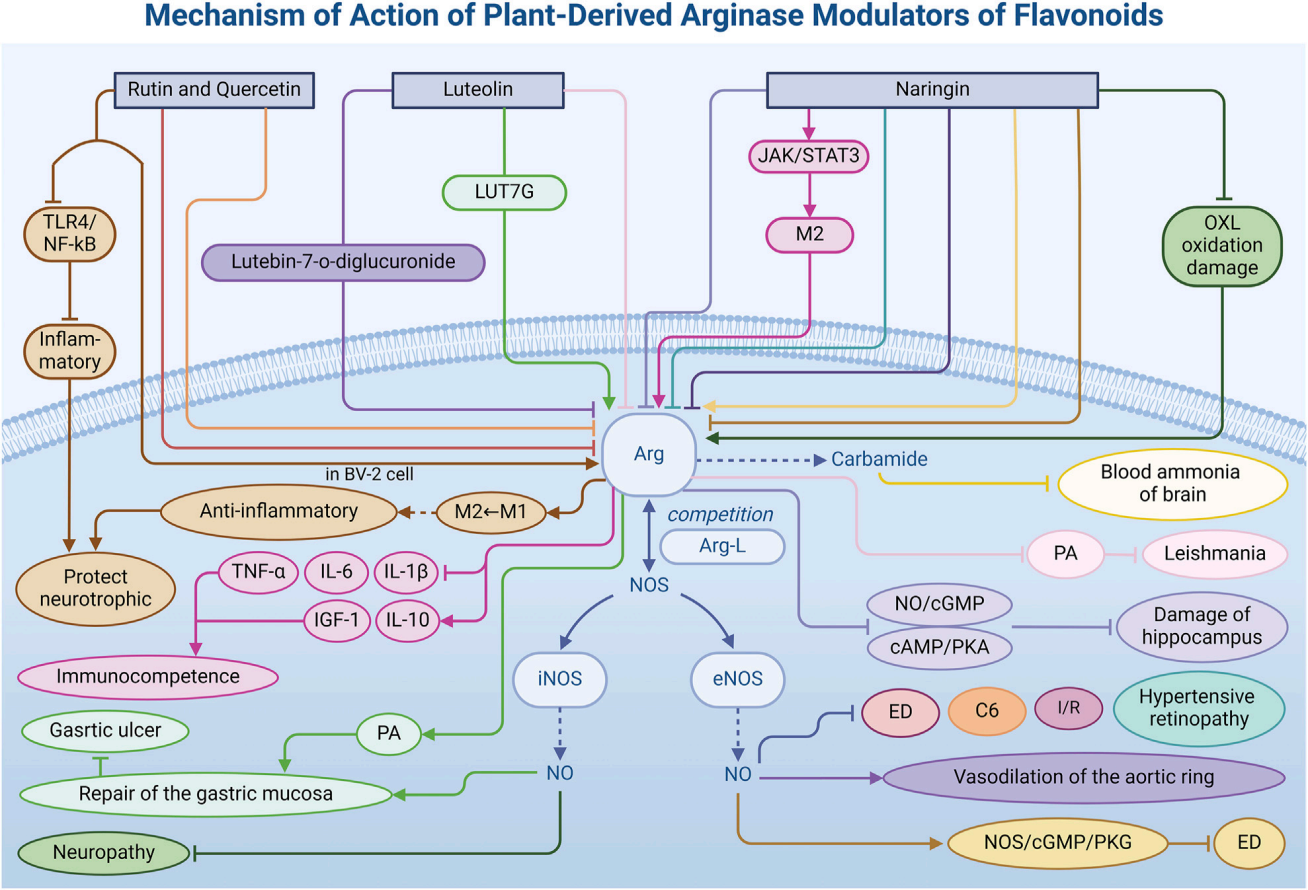
Mechanism of action of natural arginase modulators of flavonoids.

Flavonoids, a diverse class of naturally occurring organic metabolites prevalent in the plant kingdom, have garnered significant attention due to their multifaceted pharmacological properties, including antioxidant, anti-inflammatory, anticancer, and antithrombotic effects. In modulating Arg activity, flavonoids exhibit unique diversity and bidirectionality. By influencing the Arg activity, flavonoids regulate the signaling pathway related to inflammatory factors, which helps maintain the healthy development of cells. This is crucial for hypertension (Ellwood et al., 2019), neuroprotection, tumor suppression, and other physiological processes. This section delves into how specific flavonoids—rutin, its aglycone quercetin, luteolin, and naringin—modulate Arg activity (Table 1) through distinct regulatory mechanisms, contributing to the treatment of related diseases (Figure 3).

#### 3.2.1 Rutin and quercetin

Rutin **(8)** (Sharma et al., 2013), a flavonoid found in various plants, and its aglycone form quercetin **(9)**, possess significant pharmacological activities. Both metabolites effectively reduce Arg activity by inhibiting its enzymatic function or mRNA expression, thereby influencing NO metabolism. In a rat model of ED (Adefegha et al., 2018), researchers observed increased Arg activity in paroxetine-induced ED rats. Treatment with rutin and quercetin not only significantly reduced this activity, but also enhanced NO availability and improved the relaxation function of smooth muscle of the corpus cavernosum. Additionally, these flavonoids impact other key enzymes such as phosphodiesterase-5 (PDE-5), acetylcholinesterase (AChE), and angiotensin-converting enzyme (ACE), demonstrating potential in ED prevention and management. This mechanism provides a novel molecular target for ED treatment. Furthermore, the inhibitory effects of rutin and quercetin have been demonstrated in glioma treatment (da Silva et al., 2020). Investigators found that these metabolites reduced Arg mRNA expression levels when glioma C6 cells were co-cultured with microglia or indirectly contacted via conditioned medium. This regulatory effect may alter Arg-L metabolism in the tumor microenvironment, subsequently affecting tumor cell proliferation and migration. Additionally, rutin and quercetin modulate the expression of other inflammatory and growth factors, such as tumor necrosis factor (TNF), IL-6, IL-10, transforming growth factor-β (TGF-β), and insulin-like growth factor (IGF). These regulatory effects suggest that rutin and quercetin may indirectly inhibit glioma cell proliferation and migration by altering the inflammatory and immunomodulatory state within the tumor microenvironment.

On the other hand, rutin and quercetin also exhibit complex effects on Arg activation. In an immunofluorescence staining experiment (Lang et al., 2021), quercetin (50 μg/mL) pretreatment significantly increased the expression level of Arg in BV-2 cells and promoted the transformation of microglia from the pro-inflammatory M1 phenotype to the anti-inflammatory M2 phenotype. M2 microglia protects neurons by secreting anti-inflammatory components and neurotrophic factors. Moreover, quercetin further restricts the release of inflammatory factors in LPS-induced BV-2 cells by inhibiting the TLR4/NF-κB signaling pathway, providing stronger evidence for its potential as a neuroinflammatory therapeutic agent. These mechanisms have important implications for the treatment of neurodegenerative diseases such as Parkinson’s and Alzheimer’s.

#### 3.2.2 Luteolin

Luteolin **(10)**, a naturally occurring flavonoid prevalent in vegetables, fruits, botanical drugs, and other foods (Kar et al., 2024), has garnered significant attention for its medical potential as an Arg inhibitor. Exhibiting more than 50% inhibitory activity against Arg, luteolin displays substantial promise in medical applications. The study by Leticia Correa Manjolin et al. not only validated the efficacy of luteolin in treating leishmaniasis but also elucidated the mechanism by which luteolin interacts with Arg (Manjolin et al., 2013). Molecular docking studies have further revealed the interactions between luteolin and the amino acid residues involved in the formation of Mn^2+^-Mn^2+^ metal bridges at the active site of Arg. These interactions facilitate the binding and inhibition of luteolin at the enzyme’s active site (IC_50_ = 9 ± 1 μM), thereby inhibiting Arg activity and interfering with PA synthesis in Leishmania. This ultimately increases oxidative stress in parasite cells, achieving the effect of infection control.

Laboratory *in vitro* experiments have revealed that luteolin and its glucoside derivatives, particularly luteolin 7-diglucoside and luteolin 7-glucoside (Arraki et al., 2020), substantially inhibit Arg, achieving inhibition rates of 54%–83% at a concentration of 100 µM. Notably, luteolin 7-diglucoside (IC_50_ = 95.3 ± 9.5 μM) effectively counteracts the inhibitory effect of N-nitro-L-arginine-methyl ester hydrochloride (L-NAME), a nitric oxide synthase (NOS) inhibitor, on acetylcholine-induced vasodilation in isolated aortic rings, suggesting a direct enhancement of NOS activity.

However, luteolin’s action is versatile. In a gastric ulcer model (Antonisamy et al., 2016), the derivative luteolin 7-O-glucoside (LUT7G) demonstrated an opposing effect by promoting Arg activity. LUT7G facilitates the conversion of Arg-L to PAs by activating Arg. These PAs are critical for cell proliferation and tissue repair, vital for maintaining and restoring gastric mucosal health. In ethanol-induced ulcers, pretreatment with LUT7G significantly elevated Arg activity, increased competition for Arg-L between Arg and iNOS, and effectively downregulated the overproduction of NO, the product of iNOS, mitigating NO-induced damage to gastric mucosal cells. Consequently, this promoted healing and protection of the gastric mucosa. Additionally, LUT7G enhanced its protective effect on the gastric mucosa through anti-secretory, anti-inflammatory, antioxidative, and anti-apoptotic activities, while stimulating the synthesis of prostaglandin E2 (PGE2), mucus, and heat shock protein 70 (HSP-70).

#### 3.2.3 Naringin

Naringin **(11)**, a natural flavonoid prevalent in grapefruit and other citrus fruits, holds significant potential for regulating Arg activity and promoting health, particularly in the management of hypertension complications. Notably, naringin exhibits a broad inhibitory effect on Arg. Studies investigating the impact of hypertension and purine metabolism defects on hippocampal damage in rats (Akintunde et al., 2022) have revealed that alterations in Arg activity profoundly affect hippocampal integrity. Naringin treatment inhibits Arg activity, modulates the nitric oxide/cyclic guanosine monophosphate (NO/cGMP) and cyclic adenosine monophosphate/protein kinase A (cAMP/PKA) signaling pathways, supports vascular endothelial function, and preserves cell signaling, thereby protecting the hippocampus from hypertension-induced damage.

Moreover, in hypertensive rat models, exposure to L-NAME, a NOS inhibitor, and the environmental toxin bisphenol A (BPA) elevates Arg activity and decreases NO levels, contributing to ED and cataract formation. In ED (Akintunde et al., 2020a), naringin enhances erectile function by suppressing Arg activity, increasing Arg-L availability, promoting NO synthesis, and activating the NOS/cGMP/protein kinase G (PKG) signaling pathway. Concurrently, naringin regulates key enzymes implicated in ED, such as ACE, PDE-5, and AChE, which are essential for erectile function regulation. In cataract disease models (Akintunde et al., 2020b), naringin mitigates oxidative damage through its antioxidant properties and stabilizes the lipid bilayer of cell membranes. Furthermore, naringin enhances NO bioavailability, improves vascular endothelial function, and reduces the risk of hypertension-induced ocular lesions. During recovery from ischemia/reperfusion (I/R) injury (Cerkezkayabekir et al., 2017), administration of 50 mg/kg of naringin significantly reduces Arg activity in the small intestine and lungs, fostering NO production by eNOS, which prevents excessive generation of NO free radicals and associated tissue degeneration. This also reduces oxidative stress and aids tissue recovery post-I/R injury.

Additionally, naringin modulates Arg activity through diverse regulatory pathways. In NH_4_Cl-induced hyperammonemia rat models (Arumugam and Natesan, 2017), naringin enhances Arg activity, facilitates the hydrolysis of Arg-L to urea, boosts urea production, lowers blood ammonia levels, and alleviates ammonia-induced neurotoxicity. Naringin also influences M2 microglia via the Janus kinase/signal transducer and activator of transcription 3 (JAK/STAT3) signaling pathway (Li et al., 2022a), enhancing Arg expression—an M2 microglia marker—reducing inflammatory factors such as IL-1β, IL-6, and tumor necrosis factor-α (TNF-α), while promoting anti-inflammatory factors IL-10 and insulin-like growth factor-1 (IGF-1), thereby maintaining immune homeostasis. In the oxaliplatin (OXL)-induced peripheral neuropathy model (Semis et al., 2022), naringin’s role extends further. By counteracting OXL-induced oxidative stress, naringin increases intracellular Arg expression, restricts iNOS binding to Arg-L, and prevents excessive NO production, thus exerting anti-inflammatory effects and enhancing nerve blood flow and conduction. This unveils new strategies for neuroprotective and anti-inflammatory therapies.

Flavonoid Arg modulators offer significant advantages due to their multifaceted biological activities, including antioxidant, anti-inflammatory, and antitumor properties, demonstrating therapeutic potential across various diseases. The diverse chemical structures of these flavonoids enable them to exhibit varying degrees of modulation on different enzymes. However, the precise structural mechanisms by which flavonoid Arg modulators bind to Arg remain unclear. Addressing this challenge requires researchers to delve deeper into the structural characteristics of flavonoid modulators, develop comprehensive chemical structure models, and accurately select or design metabolites with high selectivity. This approach aims to facilitate more precise and reliable therapeutic applications while minimizing non-specific modulation of other enzymes or biomolecules.

### 3.3 Terpenoids: promoting effect on macrophage polarization

**FIGURE 4:**
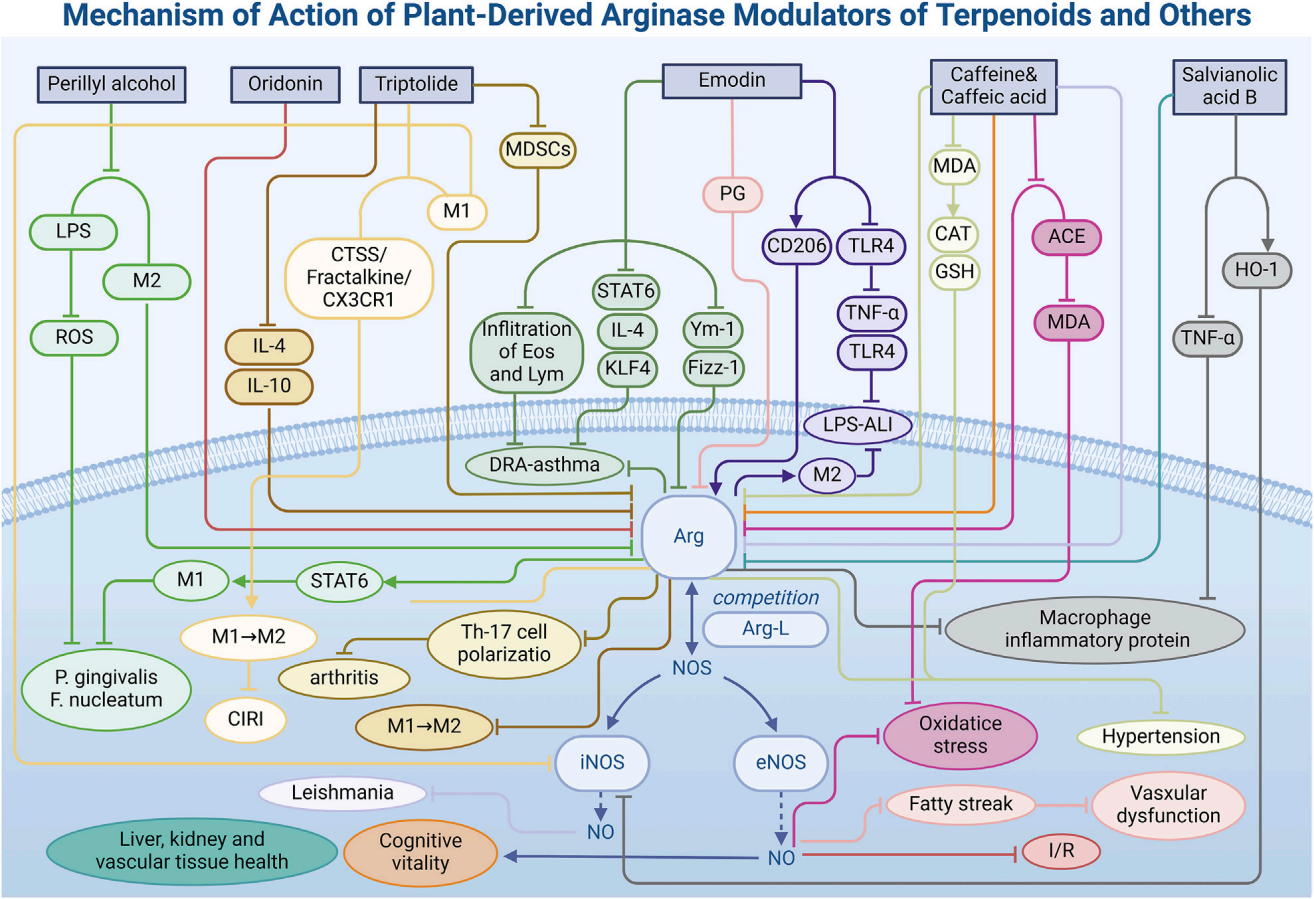
Mechanism of action of natural arginase modulators of terpenoids and other metabolites.

Terpenoids, a diverse group of natural products widely found in the biological world, are pivotal in pharmacological research due to their unique chemical structures and varied biological activities. Beyond their established antibacterial, anti-inflammatory, and antioxidant effects, terpenoids also exhibit the ability to regulate Arg activity, notably by inducing the direction of cell polarization. This section will focus on the improvement of the disease model by regulating Arg activity (Table 1) through oridonin, triptolide and perillyl alcohol under different environmental conditions (Figure 4).

#### 3.3.1 Oridonin

Oridonin **(12)**, an active diterpenoid isolated from the genus *Isodon* of the Lamiaceae family, demonstrates significant potential in treating heart diseases, attributed to its high accumulation in cellular mitochondria. Within mitochondria, oridonin modulates NO bioavailability by inhibiting Arg activity, an enzyme prevalent in cardiac mitochondria. In a rat model of MI/R injury (Zhang et al., 2019), oridonin administration markedly increased Arg-L levels in heart tissue, facilitating its binding to eNOS to produce NO, thereby effectively mitigating cardiac injury during I/R, as evidenced by a substantial reduction in infarct size. Moreover, the effects of oridonin extend beyond Arg inhibition, encompassing the regulation of glycolysis, branched-chain amino acid metabolism, tryptophan and kynurenine metabolism, and bile acid metabolism. The modulation of these metabolic pathways is intricately linked to enhanced cardiac energy metabolism, reduced oxidative stress in cardiomyocytes, and suppressed inflammatory responses. Consequently, oridonin’s cardioprotective effects are multifaceted, with Arg regulation serving as a crucial mechanism among its diverse actions. This discovery not only offers novel strategies for treating MI/R injury but also identifies valuable targets for developing new cardioprotective drugs.

#### 3.3.2 Triptolide

Triptolide **(13)**, an epoxy diterpene lactone metabolite derived from the traditional Chinese medicinal plant *Tripterygium wilfordii* Hook. f., stands as one of its principal active metabolites. Triptolide exhibits substantial anti-inflammatory and antitumor properties, particularly through the modulation of tumor-associated macrophages. In experimental models using RAW264.7 macrophages (Li et al., 2020), triptolide treatment effectively inhibited the differentiation of these cells into the M2 phenotype (IC_50_ = 25.7 nM). Real-time polymerase chain reaction (PCR) analysis revealed a marked reduction in mRNA and protein levels of Arg, an M2 macrophage marker, implying that triptolide can alter macrophage polarization by inhibiting markers like Arg. Moreover, through the assessment of T helper 2 (Th2)-type cytokine secretion, researchers observed that triptolide modulates marker expression, including Arg, by downregulating Th2 cytokines such as interleukin-4 (IL-4) and IL-10. This cytokine downregulation diminishes the immunosuppressive function of M2 macrophages, influences the metabolic context of Arg, reverses its activity and expression, thereby modulating cell polarization.

In RA patients and mouse models with arthritis (Zhao et al., 2024), researchers identified another example of triptolide modulating cellular inflammatory polarization. Triptolide treatment significantly reduced the proportion of MDSCs in peripheral blood mononuclear cells (PBMCs) of RA patients, inhibited the differentiation of MDSCs—especially the mononuclear MDSCs (M-MDSCs) subpopulation—and significantly reduced Arg protein expression in a dose-dependent manner. This effect weakened the ability of MDSCs to induce Th17 cell polarization and slowed disease progression. Additionally, triptolide treatment reduced serum IL-17 levels and the infiltration of Ly6C^+^ and IL-17^+^ cells in joint synovial tissue in ALA-induced mouse models, further confirming the immunotherapeutic effect of triptolide on arthritis.

Furthermore, in the context of anti-inflammatory and tissue repair processes during cerebral ischemia/reperfusion injury (CIRI) (Zhou et al., 2024), triptolide facilitates the polarization of microglia towards the anti-inflammatory M2 type by reducing the expression of iNOS, a marker of pro-inflammatory M1 microglia, and enhancing the expression of Arg. Concurrently, triptolide inhibits the activation of the cathepsin S/fractalkine/C-X3-C motif chemokine receptor 1 (CTSS/Fractalkine/CX3CR1) signaling pathway, thereby decreasing M1 microglia polarization and preventing apoptosis in mouse hippocampal neurons (HT-22). These two pathways demonstrate the protective effect of triptolide on neuronal cells, providing a new therapeutic strategy and potential for the prevention and treatment of CIRI.

#### 3.3.3 Perillyl alcohol

Perillyl alcohol **(14)**, a terpenoid metabolite found in the essential oils of plants such as ginger, lemon, *Perilla*, and lavender, has garnered attention for its potent antibacterial properties. In the context (Alves Figueiredo et al., 2020) of periodontal and other inflammatory diseases, perillyl alcohol has demonstrated significant *in vitro* antibacterial activity against two major Gram-negative bacteria, *Porphyromonas gingivalis* and *Fusobacterium nucleatum*. At a concentration of 100 μM, perillyl alcohol exhibited excellent cytocompatibility in the RAW 264.7 mouse macrophage cell line, showing no apparent cytotoxicity. Furthermore, it significantly inhibited the production of reactive oxygen species (ROS) induced by LPS, indicating its potential regulatory effect on the oxidative stress response of macrophages. At the molecular level, perillyl alcohol also reduces the expression of Arg in M2 macrophages, as shown by RT-qPCR, suggesting that it may modulate the STAT6 signaling pathway to influence macrophage polarization and promote the activation of M1 (pro-inflammatory) macrophages. These immunomodulatory properties of perillyl alcohol not only offer new therapeutic strategies for controlling inflammatory processes but also provide a novel perspective for the clinical treatment of inflammatory diseases such as periodontal disease. Future studies will focus on investigating the specific effects of perillyl alcohol on the progression of periodontal disease *in vivo* and its long-term regulatory impact on macrophage polarization, aiming to elucidate its molecular mechanisms and clinical application potential in treating inflammatory diseases.

The efficacy of terpenoid Arg modulators is intricately linked to the drug environment, necessitating specific conditions to regulate Arg effectively. As a result, the application of these modulators is relatively narrow and subject to significant constraints, requiring careful consideration of the interaction between terpenoid modulators and their environment. Conversely, drugs that act under specific conditions may enhance the diagnosis and treatment of particular conditions, serving as a template for precision medicine. Based on existing research, it is suggested that researchers develop diverse models to expand the application of terpenoid modulators.

### 3.4 Other metabolites

In addition to the three primary classes of natural Arg modulators previously discussed, researchers have identified additional bioactive metabolites—emodin, caffeine, and salvianolic acid B—that exhibit Arg modulatory effects (Table 1), influencing vascular health, airway inflammation, brain function, and kidney function, among others (Figure 4). These findings provide novel insights and targets for disease treatment, filling the gap in the medicinal application of natural modulators.

#### 3.4.1 Emodin

Emodin **(15)**, a naturally occurring anthraquinone derivative abundant in the rhizomes of Polygonaceae species such as *Rheum palmatum* L. and *Rheum officinale* Baill., has biological activities closely linked to the regulation of cell polarization. In a mouse model of asthma induced by a mixture of dust mites, ragweed, and *Aspergillus* fungi (DRA) (Song et al., 2018), emodin significantly mitigated airway inflammation by reducing eosinophil and lymphocyte infiltration in bronchoalveolar lavage fluid, decreasing mucus secretion, and lowering serum immunoglobulin E (IgE) production. Notably, in lung tissue, emodin markedly decreased the expression levels of Arg, Ym-1 (chitinase-like protein 3), and Fizz-1 (found in inflammatory zone 1), all of which are associated with the activation of alternatively activated macrophages (AAMs). *In vitro* studies further confirmed that emodin modulates AAM polarization by inhibiting IL-4-induced polarization and STAT6 phosphorylation, and by reducing Krüppel-like factor 4 (KLF4) expression in a dose-dependent manner.

In an LPS-induced acute liver injury (ALI) model (Ding et al., 2018), emodin significantly increased the expression of M2 macrophage markers Arg and Mannose Receptor (CD206) in mouse liver, demonstrating its potential to combat liver inflammation and alleviate liver injury by promoting M2 macrophage activation. Immunofluorescence analysis revealed increased Arg expression in emodin-treated mice, indicating healthier liver tissue and reduced inflammatory cell infiltration. The promotion of Arg expression by emodin correlated with dosage, highlighting a dose-dependent effect. Furthermore, emodin decreased the expression of pro-inflammatory factors TNF-α and IL-6 by inhibiting the TLR4 signaling pathway, presenting a potential therapeutic strategy for ALI prevention and treatment, as evidenced by the reduction of serum alanine aminotransferase (ALT) and aspartate aminotransferase (AST) levels.

Additionally, piceatannol-3-O-β-D-glucopyranoside (PG) (Kim and Ma, 2019), isolated from rhubarb, also demonstrates Arg inhibitory capacity. In a high-cholesterol diet-induced vascular disease model, PG enhanced NO production by inhibiting Arg activity and facilitating the binding of Arg-L to eNOS in vascular endothelial cells. This promotion of vasodilation and improved blood flow significantly reduced fatty streak formation induced by a high-cholesterol diet, underscoring its vital role in vascular health.

#### 3.4.2 Caffeine and caffeic acid

Caffeine **(16)** and caffeic acid **(17)** are two bioactive metabolites prevalent in various plants, notably in coffee beans, tea leaves, and cocoa beans. These metabolites function as central nervous system stimulants with potential beneficial effects on brain function. Particularly noteworthy is their modulatory impact on Arg, an enzyme integral to brain health and function. Study (Oboh et al., 2021) has demonstrated that both caffeine and caffeic acid independently reduce Arg activity in the rat brain and cortex, with their combination amplifying this inhibitory effect. This mechanism increases the availability of Arg-L for the NOS pathway, thereby enhancing NO production, which is crucial for memory and cognitive function improvement.

Moreover, the antioxidant properties of caffeine and caffeic acid, along with their inhibitory effects on AChE and ADA activities (Akomolafe, 2017), underscore their potential to enhance brain function. In the L-NAME-induced hypertensive rat model, pretreatment with these metabolites significantly reduced Arg and ACE activities, while also providing cardiovascular protection by mitigating oxidative stress. This was achieved by increasing the bioavailability of nitric oxide and its derivatives (NOx) and reducing malondialdehyde (MDA) content. In addition, Odunayo et al. identified another mechanism by which caffeic acid relieves hypertension in cyclosporin-induced hypertensive rats (Agunloye et al., 2019). Caffeic acid not only increases NO bioavailability by decreasing Arg activity but also significantly reduces MDA levels while increasing the activity of catalase (CAT) and GSH. These actions contribute to its strong antioxidant effects and role in reducing hypertension. These studies provide evidence for the use of caffeine and caffeic acid to influence brain function and improve cognitive health through the pathway of regulating Arg activity.

Norhan et al. further verified the effects of caffeic acid on hyperglycemia, hyperlipidemia, cognitive dysfunction, and anxiety-like behavior induced by a high-fat diet (El-Sayed et al., 2024). In behavioral tests, rats treated with caffeic acid exhibited better spatial learning and memory in the Morris water maze test and reduced anxiety-like behavior in the light-dark box test. Biochemical analysis showed that caffeic acid significantly reduced oxidative stress markers (such as MDA and NO), increased antioxidant enzyme activity (such as GSH, superoxide dismutase (SOD), and glutathione S-transferase (GST)), and decreased inflammatory markers (such as IL-1β, IL-2, TNF-α, and IFN-γ). This suggests that caffeic acid reduces inflammation-related brain damage. Histological examination revealed that caffeic acid activated the Wnt/β-catenin pathway and inhibited the activity of glycogen synthase kinase-3β (GSK-3β), thereby promoting brain-derived neurotrophic factor (BDNF) expression and improving cognitive function.

Caffeic acid also shows promise in treating hepatic leishmaniasis. Andreza et al. investigated the biochemical properties of leishmanial Arg and found that caffeic acid could serve as a potential therapeutic strategy to reduce parasite growth and infectivity by inhibiting Arg (Garcia et al., 2019). The results showed that caffeic acid had a strong inhibitory effect on Arg activity between 37°C and 55°C (56.98% and 71.48%, respectively) and significantly increased NO production in infected macrophages. *In vivo* experiments demonstrated that caffeic acid was effective and highly selective against both the promastigote and intracellular amastigote forms of Leishmania. This provides a novel approach for treating leishmaniasis using caffeic acid as a natural phenolic compound.

#### 3.4.3 Salvianolic acid B

Salvianolic acid B **(18)** is a water-soluble metabolite extracted from the roots and rhizomes of *Salvia miltiorrhiza* Bunge, commonly known as Danshen. Renowned for its potent antioxidant properties, it ranks among the most powerful natural antioxidants known. The regulatory effects of salvianolic acid B on Arg activity are tissue-specific, varying with sites of action. In RAW 264.7 macrophage cells (Joe et al., 2012), salvianolic acid B exhibits anti-inflammatory effects by inhibiting LPS-induced TNF-α production and enhancing heme oxygenase-1 (HO-1) activity in a dose-dependent manner. This results in the inhibition of NOS expression and an increase in Arg activity. Conversely, salvianolic acid B inhibits Arg activity in the liver (IC_50_ = 1.44 mg/L), kidney, and vascular tissues of mice (Abdelkawy et al., 2017), leading to elevate NO production in the mouse aorta, which is closely associated with endothelium-dependent vasodilation. The experiment not only demonstrated the ability of salvianolic acid B to regulate Arg, but also emphasized the decisive influence of tissue environmental conditions on Arg function, providing new thinking for the selection of tissue models for subsequent studies.

However, pharmacokinetic studies reveal that salvianolic acid B exhibits low bioavailability across various animal models, necessitating further research to enhance its bioavailability and efficacy. Additionally, the impact of salvianolic acid B on the cytochrome P450 enzyme system remains contentious, warranting further investigation. In conclusion, salvianolic acid B, as an Arg modulator, not only plays a role in regulating Arg-L metabolism but may also protect vascular health through multiple mechanisms. Future studies should focus on optimizing its pharmacokinetic properties and evaluating its potential for clinical application.

## 4 Evaluation of pharmacokinetic characteristics of natural metabolites

To fully realize the therapeutic potential of natural Arg modulators in clinical applications, a comprehensive understanding of their pharmacokinetic properties (Table 2) is crucial to ensure optimal absorption, distribution, and safe elimination in the human body. Studies employing both human and rat models have consistently revealed significant variations among different natural metabolites in key pharmacokinetic parameters, including bioavailability, retention time, and tissue distribution profiles. These observed differences in pharmacokinetic behavior not only directly impact their efficacy as therapeutic agents but also highlight the distinct challenges inherent in transforming natural metabolites into clinically viable pharmaceuticals.

**TABLE 2:** Pharmacokinetic data of natural metabolites.

Natural metabolites	Subject	Weight	Method	Dose	C_max_	AUC_0-∝_	T_max_	T_half_	Ref.
Resveratrol	Colon cancer patient	—	Oral administration	5 g/d for 8 days	1942 ng/mL	—	1.5–2 h	—	Patra et al. (2021)
Curcumin	Pancreatic cancer patients	—	Oral administration	Oral pills every day for 8 weeks	7 nM	—	6 h	—	Patra et al. (2021)
EGCG-3-gallate	Healthy volunteers	—	Oral administration	73 mg (single-dose)	0.71 μM	—	1.5–2.5 h	4.9–5.5 h	Chow and Hakim (2011)
Rutin	Healthy volunteers	—	Oral administration	200 mg/d on Days 4, 6, 8 and 10	0.3 ± 0.3 μg/mL	2.5 ± 2.2 μg·h/mL (AUC_0–24 h_)	7.0 ± 2.9 h	11.8 ± 3.1 h	Graefe et al. (2001)
Luteolin	Adult male Sprague-Dawley rat	—	Intravenous injection	10 mg/kg (single-dose)	7.47 ± 3.78 μg/mL	261 ± 33 μg·min/mL	5 min	78 ± 14 min	Lin et al. (2015)
Luteolin	Adult male Sprague-Dawley rat	—	Oral administration	100 mg/kg (single-dose)	3.07 ± 0.72 μg/mL	611 ± 89 μg·min/mL	5 min	132 ± 12 min	Lin et al. (2015)
Naringin	Healthy volunteers	55–80 kg	Oral administration	135 mg (single-dose)	2009.51 ng/mL	9,424.52 ng·h/mL	3.67 h	2.31 h	Kanaze et al. (2007)
Oridonin	Kunming mice	25 ± 2 g	Intravenous injection via the tail vein	20 mg/kg (single-dose)	—	AUC_0–12 h_: 26.01 mg·h/L (plasma) 97.78 mg·h/L (heart) 58.66 mg·h/L (liver)	—	1.74 h	Gao et al. (2008)
Triptolide	Male Sprague-Dawley rat	200–300 g	Oral administration	0.4 mg/kg (single-dose)	5.16 ± 1.36 ng/mL	2.60 ± 0.58 ng·h/mL	0.21 ± 0.08 h	0.44 ± 0.08 h	Xu et al. (2018)
Perillyl alcohol	Male Sprague-Dawley rat	250 ± 20 g	Intravenous injection via the tail vein	65 mg/kg (single-dose)	—	4,944.2 ± 24.4 mg/L/min	—	81.65 ± 1.93 min	Hua et al. (2008)
Emodin	Male Sprague-Dawley rat	320 ± 25 g	Oral administration	20 g/kg (single-dose)	0.478 mg/L	1.236 mg·h/L (AUC_0–12 h_)	28.02 min	13.2 min	Gong et al. (2011)
Caffeic acid	Male Sprague-Dawley rat	180–200 g	Intragastric administration	1.5 mL (Cohosh extract)/100 g (single-dose)	2055.05 ± 501.00 mg/L	5,745.29 ± 1,426.55 mgh/L	0.60 ± 0.25 h	4.14 ± 0.50 h	Wu et al. (2020)
Salvianolic acid B	Healthy volunteers	≥50 kg	Intravenous injection	75, 150 or 300 mg (single-dose)	75 mg: 3,431 ± 781 ng/mL 150 mg: 8,646 ± 2,580 ng/mL 300 mg: 15,925 ± 6,082 ng/mL	75 mg: 3,660 ± 995 ng·h/mL 150 mg: 10,404 ± 4,025 ng·h/mL 300 mg: 17,095 ± 8,113 ng·h/mL	75 mg: 0.95 ± 0.12 h 150 mg: 0.97 ± 0.09 h 300 mg: 0.95 ± 0.12 h	75 mg: 0.67 ± 0.27 h 150 mg: 1.45 ± 0.50 h 300 mg: 2.13 ± 0.73 h	Cheng et al. (2023)

To comprehensively illustrate the challenges associated with natural metabolites in drug metabolism, three representative case studies are presented based on the completeness of their pharmacokinetic data and the significance of the issues they highlight. The first case involves rutin (Graefe et al., 2001), whose pharmacokinetic profile demonstrates a low maximum plasma concentration (C_max_ = 0.3 ± 0.3 μg/mL) and a prolonged time to reach peak concentration (T_max_ = 7.0 ± 2.9 h), indicating slow absorption and delayed onset of action. However, its relatively high systemic exposure, as evidenced by the area under the concentration-time curve from 0 to 24 h (AUC_0-24_ = 2.5 ± 2.2 μg h/mL), coupled with a long half-life (T_half_ = 11.8 ± 3.1 h), suggests potential for sustained regulatory effects. Future research should focus on optimizing administration methods and enhancing drug transport mechanisms to improve rutin’s bioavailability in clinical applications. Another notable example is salvianolic acid B (Cheng et al., 2023). Under intravenous injection conditions, its pharmacokinetic profile is characterized by high C_max_ (75 mg: 3,431 ± 781 ng/mL, 150 mg: 8,646 ± 2,580 ng/mL, 300 mg: 15,925 ± 6,082 ng/mL) and AUC_0-∞_ (75 mg: 3,660 ± 995 ng·h/mL, 150 mg: 10,404 ± 4,025 ng·h/mL, 300 mg: 17,095 ± 8,113 ng·h/mL), as well as short T_max_ (75 mg: 0.95 ± 0.12 h, 150 mg: 0.97 ± 0.09 h, 300 mg: 0.95 ± 0.12 h) and T_half_ (75 mg: 0.67 ± 0.27 h, 150 mg: 1.45 ± 0.50 h, 300 mg: 2.13 ± 0.73 h). These attributes suggest that salvianolic acid B possesses a rapid onset of action, making it advantageous for therapeutic scenarios that require immediate efficacy. However, its rapid clearance rate raises concerns regarding the potential for frequent dosing-related toxicity in long-term treatment regimens. Future research should focus on developing strategies to mitigate these challenges while preserving its therapeutic efficacy. Moreover, differences in tissue distribution represent a critical area for improvement in drug development. For example, the pharmacokinetic profile of oridonin (Gao et al., 2008) demonstrates varying AUC_0-12_ in plasma (26.01 mg·h/L), heart (97.78 mg·h/L), and liver (58.66 mg·h/L), indicating significant differences in tissue exposure levels. This highlights the need for targeted drug delivery systems to ensure that therapeutic agents achieve optimal concentrations at the site of action while minimizing off-target effects. Future research should prioritize the development of tissue-specific carriers tailored to the unique characteristics of human tissue environments, thereby enhancing the targeting efficiency of drug molecules and preserving the homeostasis of non-target tissues.

Beyond their inherent pharmacokinetic attributes, the administration routes of natural flavonoids profoundly shape their pharmacokinetics and ultimately influence their pharmacodynamic outcomes. For example, luteolin-7-O-glucoside was found to exhibit a limited bioavailability of just 10% ± 2% when administered orally at a dose of 1 g/kg (Lin et al., 2015). In stark contrast, intravenous administration of the same compound (10 mg/kg) bypassed gastrointestinal processing and facilitated its direct entry into systemic circulation, notably without the generation of the metabolite luteolin. Conversely, oral administration of luteolin (100 mg/kg) achieved a higher bioavailability of 26% ± 6%, characterized by a biphasic pharmacokinetic pattern, indicative of enterohepatic recirculation. Interestingly, luteolin-7-O-glucoside undergoes hydrolysis via β-glucosidase in the gastrointestinal tract before absorption, which contributes to its notably high conversion efficiency to luteolin, as reflected by a conversion rate of 48.78% ± 0.12% (AUC metabolite/parent drug ratio) in plasma. This differential metabolic processing significantly influences the systemic exposure of the parent flavonoid and its metabolites, while also potentially altering its antioxidant and anti-inflammatory activities. These findings highlight the pivotal role of administration routes in modulating therapeutic outcomes and optimizing the efficacy of natural flavonoid-based interventions.

In summary, to bridge the existing knowledge gaps in natural metabolite-based drug development, future research should prioritize the comprehensive elucidation of pharmacokinetic profiles in human models. Through systematic investigation and comparative analysis of these pharmacokinetic characteristics, researchers will be able to establish more precise and targeted research frameworks. Such methodological advancements will significantly contribute to the development of safer, more effective, and tissue-specific therapeutic agents derived from natural sources, thereby accelerating the clinical translation of natural Arg modulators and enhancing their therapeutic potential.

## 5 Conclusion and prospects

### 5.1 Advances in natural arginase modulators

In recent years, Arg modulators have garnered significant attention due to their diverse regulatory mechanisms. By specifically targeting Arg, these agents interfere with its physiological role in the urea cycle and modulate Arg-induced tumor growth and immunosuppression under pathological conditions. Natural Arg modulators, in particular, have demonstrated superior biocompatibility (Canali et al., 2010; Bygd et al., 2017) and a lower risk of side effects (Kumari and Bansal, 2021; Erens et al., 2023; Bunch et al., 2022) compared to chemically synthesized modulators in experimental studies. Categorized into three groups based on their molecular structures—polyphenols, flavonoids, and terpenoids—these modulators have exhibited remarkable therapeutic effects in treating inflammatory diseases such as hypertension and its associated complications, cancer and mental disorders.

For instance, resveratrol can effectively occupy and block the active site of Arg-L through signaling pathways such as PI3K-Akt (Chen et al., 2014) and CaMKII (Trieu Dieu Linh et al., 2016) by forming hydrogen bonds with Arg-L amino acid residues and regulate the expression level of Arg-L. This action positively impacts vasodilation and inhibits vascular cell proliferation, offering a novel perspective for treating hypertension and its complications. Furthermore, natural Arg modulators have demonstrated significant anti-inflammatory effects concerning neuroprotection and intestinal health. Naringin (Li et al., 2022a), for example, facilitates the transition of M1 macrophages to M2 macrophages via the JAK/STAT3 signaling pathway, which enhances the expression of Arg as a marker of M2 macrophages, and mitigates the pro-inflammatory traits of M1 macrophages, thereby conferring neuroprotective benefits. Additionally, Curcumin (Islam et al., 2021) addresses WAT inflammation by modulating intestinal flora composition, preserving intestinal barrier integrity, inhibiting the STAT6 signaling pathway, and reducing Arg expression. Researchers have also identified that certain natural Arg modulators can reverse Arg activity and expression by altering the reaction environment. As proof, triptolide (Li et al., 2020) downregulates Th2-type cytokines such as IL-4 and IL-10, diminishes the immunosuppressive function of M2 macrophages, and alters the metabolic context of Arg-L, achieving precise regulation of cell polarization. These discoveries not only deepen our understanding of the regulatory effects of natural Arg modulators but also furnish more precise strategies for disease treatment.

### 5.2 Challenges in researching natural arginase modulators

Despite notable advancements in the research of plant-based Arg modulators, challenges persist due to their diverse sources and intricate molecular structures, which complicate the comprehensive elucidation of their mechanisms of action and the innovation of drug delivery systems. These difficulties constrain the effectiveness and specificity of drug design. To address these challenges, a range of strategies has been implemented, including precise experimental design, molecular structure optimization, and delivery system innovation. In understanding their mechanisms of action, detailed analyses of the molecular recognition processes (Azizian et al., 2010; Wang et al., 2018) of natural Arg modulators offer a robust theoretical basis for drug design. Such refined studies are crucial for molecular docking analyses of modulators and enzymes. However, existing research often disproportionately emphasizes the pharmacological performance of natural metabolites like rutin and quercetin while neglecting the intricate details of their binding mechanisms with targets. This bias hinders the accurate understanding of modulator–Arg interactions, affecting the precision and efficacy of drug design. In addition, the shortcomings of the current research design of natural modulators also limit the in-depth understanding of the mechanism of action. For example, many studies rely on animal models with relatively small sample sizes, and there is a distinct lack of clinical sample validation in humans, which may render experiments insufficiently representative of the wide range of physiological responses observed in the population, meaning that the translational potential of these findings for human medicine remains uncertain. Especially for salvianolic acid B (Joe et al., 2012) with a small number of cases, the lack of human clinical samples leads to a great gap in its medicinal value. At the same time, the duration of most of the trials was short, such as the EGCG treatment of leishmaniasis experiment (Goncalves dos Reis et al., 2013; Abdelkawy et al., 2017), the inhibitor and substrate reaction lasted only 15 min. Although inhibition of Arg by EGCG can be verified by urea analysis, this experiment cannot accurately assess long-term effects and potential chronic toxicity. The pharmacokinetics of some natural Arg-L modulators, such as perillyl alcohol and caffeine and caffeic acid, in humans are significantly less than those of synthetic modulators (Blaszczyk et al., 2020), especially in the detailed data of Arg activity and its regulation over time. This knowledge gap prevents a full understanding of how these modulators behave in the body and their overall therapeutic potential. These limitations due to experimental design need to be addressed through more targeted and in-depth studies.

To address these challenges, researchers can employ computational chemistry (da Silva et al., 2019a; da Silva et al., 2019b; Lisi et al., 2017) and bioinformatics (Siavelis et al., 2016; Buscher et al., 2017; Hemmat et al., 2020) to conduct thorough analyses of structural features, accurately predicting high-resolution three-dimensional structures of Arg binding sites and simulating dynamic binding processes. This methodology enhances the design of more targeted and effective drug molecules. In advancing drug delivery systems, enhancing bioavailability and minimizing side effects are paramount. Given the significant biochemical disparities between cellular, tissue homogenate, and human environments, the design of natural Arg modulators must be adapted to align with actual drug effects during delivery. For instance, EGCG-3-gallate’s *in vivo* uptake and distribution are limited due to its molecular characteristics, such as high molecular weight and phenolic groups. When administered orally, the plasma concentration of EGCG-3-gallate in humans is significantly lower than levels demonstrating antitumor activity *in vitro*, indicating low bioavailability and reduced efficacy. To mitigate this, cationic chitosan nanoparticles and dipolyelectrolyte cation–anion nanoparticles have been employed as carriers, successfully increasing EGCG-3-gallate’s serum bioavailability by up to threefold. Moreover, utilizing gold and silver inorganic nanoparticles (Lan et al., 2024; Jackson and Dietrich, 2024; Zhang et al., 2024; Wu et al., 2024; Peng et al., 2024) as carriers not only protects EGCG-3-gallate from degradation but also enhances its absorption. These strategies improve the efficacy and safety of natural Arg modulators and furnish novel methodologies for future drug design and delivery. Moreover, addressing knowledge gaps in drug combination therapies remains a critical priority for researchers. The strategic combination of drugs offers the potential to overcome the limitations posed by the weak or incomplete therapeutic effects of natural metabolites when used independently. For instance, a recent study demonstrated that combining an Arg inhibitor (L-Norvaline) with ADI-PEG 20 (Ye et al., 2023) significantly enhanced tumor-infiltrating CD8^+^ T cells and CCR7^+^ dendritic cells, thereby potentiating robust anti-cancer immune responses. This example underscores the potential of leveraging natural metabolism modulators in tandem with established therapeutics to achieve synergistic, higher-order therapeutic outcomes. Future exploration into such combinatorial strategies could unlock new opportunities for precision medicine.

### 5.3 Future directions for arginase modulator research

To further explore the potential of natural Arg modulators, future research should focus on elucidating their molecular mechanisms and conducting comprehensive preclinical and clinical studies to validate their therapeutic potential. Researchers are encouraged to design diverse experimental conditions to fully understand how different environmental factors (Zanier et al., 2014) influence activity variations and contribute to study outcomes. For instance, in studies on periodontal pathogens, perillyl alcohol did not affect Arg expression in macrophages polarized without stimulatory factors. However, when macrophages were stimulated with IL-4 and transitioned to M2 polarization, perillyl alcohol significantly reduced Arg expression, potentially by inhibiting the STAT6 signaling pathway, thereby affecting macrophage polarization and exerting its regulatory role. Based on this, we highlight the concern about the different mechanisms of action that Arg modulators may exhibit in different situations, emphasizing the need for diversified research to refine the therapeutic application of natural Arg modulators.

Further clinical investigations are essential to establish a robust scientific foundation for validating the therapeutic efficacy of these modulators. A critical aspect of this process involves precise dose optimization, where accurately defining the therapeutic window is paramount to achieving an optimal balance between efficacy and safety (Dedkova and Blatter, 2009). For instance, while triptolide demonstrates therapeutic potential, its hepatic metabolism generates electrophilic groups, free radicals, and ROS, potentially leading to hepatotoxicity and narrowing its therapeutic safety window. To address these challenges, researchers must implement stringent dosage controls and pursue chemical structure optimization strategies to eliminate or modify functional groups that interact with hepatotoxic targets, thereby mitigating toxicity risks and enhancing therapeutic precision. Moreover, it is imperative for researchers to thoroughly understand the physiological (Kararli, 1995; Glanz et al., 2018; Zimmerman et al., 2021) and metabolic (Lubinski and Thompson, 1993; Sanoh and Ohta, 2014; Glanz et al., 2018) differences between animal models and human systems. When conducting clinical trials under ethical guidelines, careful consideration must be given to potential variations in efficacy, dosage requirements, and toxicity profiles (Brent, 2004; Vahter et al., 2007; Li et al., 2022a) resulting from the complex human internal environment. As the toxicological database for human models continues to expand and improve, researchers will gain deeper insights into the therapeutic advantages of natural metabolites. This growing knowledge base will further elucidate the safety profile and clinical feasibility of natural metabolite-based treatments, ultimately advancing their application in human medicine.

Additionally, identifying natural Arg modulators (Pudlo et al., 2017; Girard-Thernier et al., 2015; Muller et al., 2022) remains a significant research direction. When evaluating novel extracts or metabolites, researchers can systematically compare natural modulators with chemically synthesized ones by constructing biological network models to verify the potential advantages of natural-origin modulators. This comparison should encompass IC_50_ and inhibition constant (K_i_) values of the extract or pure metabolite, comparing them with those of standard reference modulators. Concurrently, researchers should screen for Arg modulators with isoform selectivity, aiming to identify metabolites (Pudlo et al., 2017) that distinguish between the two Arg isoforms, Arg 1 and Arg 2, to aid in developing new drugs with specific biological activities and therapeutic potential.

In conclusion, while our preliminary studies underscore the potential of natural Arg modulators in disease treatment, a comprehensive understanding of their mechanisms of action and long-term safety remains imperative. As the diversity and clinical applications of these modulators expand, there is a justified expectation that they will assume a more pivotal role in the realms of precision and personalized medicine.

## Glossary

Arg: Arginase
Arg-L: Arginine
Arg1: arginase 1
Arg2: arginase 2
eNOS: endothelial Nitric Oxide Synthase
iNOS: inducible Nitric Oxide Synthase
ED: Erectile Dysfunction
CaMKII: Ca^2+^/Calmodulin-Dependent Protein Kinase II
PI3K-AKT: Phosphatidylinositol 3-Kinase - Protein Kinase B Signal Pathway
hPASMC: Human Pulmonary Artery Smooth Muscle Cells
nLDL: Natural Low-Density Lipoprotein
SIRT1: NAD-dependent deacetylase sirtuin-1
RA: Rheumatoid Arthritis
LPS: Lipopolysaccharides
M1: Classic Activated Macrophages
M2: Alternatively Activated Macrophages
EAE: Experimental Autoimmune Encephalomyelitis
EB: Evans Blue
BBB: Blood-Brain Barrier
IL-1β: Interleukin-1β
ADA: Adenosine Deaminase
WAT: White Adipose Tissue
IL-6: Interleukin-6
MDSCs: Myeloid-Derived Suppressor Cells
DMH: 1,2-Dimethylhydrazine
GSH: Glutathione
TGF-β1: Transforming Growth Factor-β1
HES-1: Hes Family BHLH Transcription Factor 1
PA: Polyamine
PAs: Polyamines
PDE-5: Phosphodiesterase-5
AChE: Acetylcholinesterase
ACE: Angiotensin-converting enzyme
TNF: Tumor Necrosis Factor
IL-10: Interleukin-10
TGF-β: Transforming Growth Factor-β
ROS: Reactive Oxygen Species
IGF: Insulin-like Growth Factor
PGE2: Prostaglandin E2
HSP-70: Heat Shock Protein 70
NO/cGMP: Nitric Oxide/cyclic Guanosine Monophosphate
cAMP/PKA: cyclic Adenosine Monophosphate/Protein Kinase A
L-NAME: N-Nitro-L-Arginine-Methyl Ester hydrochloride
BPA: Environmental toxin Bisphenol A
NOS/cGMP/PKG: Nitric Oxide Synthase/ cyclic Guanosine Monophosphate/ Protein Kinase G
I/R: Ischemia/Reperfusion
STAT3: Signal Transducer and Activator of Transcription 3
JAK/STAT3: Janus kinase/signal transducer and activator of transcription 3
TNF-α: Tumor Necrosis Factor-α
IGF-1: Insulin-like Growth Factor-1
OXL: Oxaliplatin
MI/R: Myocardial Ischemia/Reperfusion
PCR: Polymerase Chain Reaction
Th2: T helper 2 cell
IL-4: Interleukin-4
CIRI: Cerebral Ischemia/Reperfusion Injury
CTSS/Fractalkine/CX3CR1: Cathepsin S/ Fractalkine/ Recombinant Chemokine C-X3-C-Motif Receptor 1
HT-22: Mouse Hippocampal Neurons
STAT6: Signal Transducer and Activator of Transcription 6
DRA: A mixture of Dust mites, Ragweed, and Aspergillus fung
IgE: Immunoglobulin E
AAMs: Alternatively Activated Macrophages
KLF4: Krüppel-Like Factor 4
ALI: Acute Liver Injury
CD206: Mannose Receptor
TLR4: Toll-Like Receptor 4
ALT: Alanine Aminotransferase
AST: Aspartate Aminotransferase
MDA: malondialdehyde
NOS: nitric oxide synthase
NF-κB: nuclear factor kappa B

## References

[B1] Abdelkawy K. S. Lack K. Elbarbry F. (2017). Pharmacokinetics and pharmacodynamics of promising arginase inhibitors. Eur. J. Drug Metab. Pharmacokinet. 42, 355–370. 10.1007/s13318-016-0381-y 27734327

[B2] Adefegha S. A. Oyeleye S. I. Dada F. A. Olasehinde T. A. Oboh G. (2018). Modulatory effect of quercetin and its glycosylated form on key enzymes and antioxidant status in rats penile tissue of paroxetine-induced erectile dysfunction. Biomed. Pharmacother. 107, 1473–1479. 10.1016/j.biopha.2018.08.128 30257364

[B3] Adewale O. O. Bakare M. I. Adetunji J. B. (2021). Mechanism underlying nephroprotective property of curcumin against sodium nitrite-induced nephrotoxicity in male Wistar rat. J. Food Biochem. 45, e13341. 10.1111/jfbc.13341 32648259

[B4] Agunloye O. M. Oboh G. Ademiluyi A. O. Ademosun A. O. Akindahunsi A. A. Oyagbemi A. A. (2019). Cardio-protective and antioxidant properties of caffeic acid and chlorogenic acid: mechanistic role of angiotensin converting enzyme, cholinesterase and arginase activities in cyclosporine induced hypertensive rats. Biomed. Pharmacother. 109, 450–458. 10.1016/j.biopha.2018.10.044 30399581

[B5] Akintunde J. K. Abinu O. S. Taiwo K. F. Sodiq R. A. Folayan A. D. Ate A. D. (2022). Disorders of hippocampus facilitated by hypertension in purine metabolism deficiency is repressed by naringin, a bi-flavonoid in a rat model via NOS/cAMP/PKA and DARPP-32, BDNF/TrkB pathways. Neurotoxic. Res. 40, 2148–2166. 10.1007/s12640-022-00578-4 36098940

[B6] Akintunde J. K. Akintola T. E. Aliu F. H. Fajoye M. O. Adimchi S. O. (2020a). Naringin regulates erectile dysfunction by abolition of apoptosis and inflammation through NOS/cGMP/PKG signalling pathway on exposure to Bisphenol-A in hypertensive rat model. Reprod. Toxicol. 95, 123–136. 10.1016/j.reprotox.2020.05.007 32428650

[B7] Akintunde J. K. Akintola T. E. Hammed M. O. Amoo C. O. Adegoke A. M. Ajisafe L. O. (2020b). Naringin protects against bisphenol-A induced oculopathy as implication of cataract in hypertensive rat model. Biomed. Pharmacother. 126, 110043. 10.1016/j.biopha.2020.110043 32172062

[B8] Akinyemi A. J. Onyebueke N. Faboya O. A. Onikanni S. A. Fadaka A. Olayide I. (2017). Curcumin inhibits adenosine deaminase and arginase activities in cadmium-induced renal toxicity in rat kidney. J. Food Drug Anal. 25, 438–446. 10.1016/j.jfda.2016.06.004 28911688 PMC9332529

[B9] Akomolafe S. F. (2017). The effects of caffeine, caffeic acid, and their combination on acetylcholinesterase, adenosine deaminase and arginase activities linked with brain function. J. Food Biochem. 41, e12401. 10.1111/jfbc.12401

[B10] Akomolafe S. F. Olasehinde T. A. Adewale O. O. Ajayi O. B. (2021). Curcumin improves biomolecules associated with renal function and attenuates oxidative injury and histopathological changes in potassium-induced toxicity in rats' kidney. Biol. Trace Elem. Res. 199, 197–204. 10.1007/s12011-020-02113-y 32277397

[B11] Akomolafe S. F. Olasehinde T. A. Oyeleye S. I. Aluko T. B. Adewale O. O. Ijomone O. M. (2020). Curcumin administration mitigates cyclophosphamide-induced oxidative damage and restores alteration of enzymes associated with cognitive function in rats' brain. Neurotoxic. Res. 38, 199–210. 10.1007/s12640-020-00205-0 32405958

[B12] Alves Figueiredo R. D. Ortega A. C. Gonzalez Maldonado L. A. de Castro R. D. Avila-Campos M. J. Rossajunior C. (2020). Perillyl alcohol has antibacterial effects and reduces ROS production in macrophages. J. Appl. Oral Sci. 28, e20190519. 10.1590/1678-7757-2019-0519 32348444 PMC7185983

[B13] Antonisamy P. Subash-Babu P. Albert-Baskar A. Alshatwi A. A. Aravinthan A. Ignacimuthu S. (2016). Experimental study on gastroprotective efficacy and mechanisms of luteolin-7-O-glucoside isolated from Ophiorrhiza mungos Linn. in different experimental models. J. Funct. Foods 25, 302–313. 10.1016/j.jff.2016.06.003

[B14] Arraki K. Totoson P. Attia R. Zedet A. Pudlo M. Messaoud C. (2020). Arginase inhibitory properties of flavonoid compounds from the leaves of Mulberry (Morus alba, Moraceae). J. Pharm. Pharmacol. 72, 1269–1277. 10.1111/jphp.13297 32496585

[B15] Arumugam R. Natesan V. (2017). Urea cycle pathway targeted therapeutic action of naringin against ammonium chloride induced hyperammonemic rats. Biomed. Pharmacother. 94, 1028–1037. 10.1016/j.biopha.2017.08.028 28813782

[B16] Azizian H. Bahrami H. Pasalar P. Amanlou M. (2010). Molecular modeling of helicobacter pylori arginase and the inhibitor coordination interactions. J. Mol. Graph. Modell. 28, 626–635. 10.1016/j.jmgm.2009.12.007 20080052

[B17] Bak A. Kos J. Degotte G. Swietlicka A. Strharsky T. Pindjakova D. (2023). Towards arginase inhibition: hybrid SAR protocol for property mapping of chlorinated N-arylcinnamamides. Int. J. Mol. Sci. 24, 3611. 10.3390/ijms24043611 36835023 PMC9968098

[B18] Bhatta A. Yao L. Xu Z. M. Toque H. A. Chen J. J. Atawia R. T. (2017). Obesity-induced vascular dysfunction and arterial stiffening requires endothelial cell arginase 1. Cardiovasc. Res. 113 (13), 1664–1676. 10.1093/cvr/cvx164 29048462 PMC6410953

[B19] Blaszczyk R. Brzezinska J. Dymek B. Stanczak P. S. Mazurkiewicz M. Olczak J. (2020). Discovery and pharmacokinetics of sulfamides and guanidines as potent human arginase 1 inhibitors. ACS Med. Chem. Lett. 11 (4), 433–438. 10.1021/acsmedchemlett.9b00508 32292546 PMC7153016

[B20] Bollenbach A. Bakker S. J. L. Tsikas D. (2019). GC–MS measurement of biological N^G^hydroxylarginine, a stepmotherly investigated endogenous nitric oxide synthase substrate and arginase inhibitor. Amino Acids 51, 627–640. 10.1007/s00726-018-02695-x 30610471

[B21] Boonpiyathad T. Sözener Z. C. Satitsuksanoa P. Akdis C. A. (2019). Immunologic mechanisms in asthma. Semin. Immunol. 46, 101333. 10.1016/j.smim.2019.101333 31703832

[B22] Bounaama A. Djerdjouri B. Laroche-Clary A. Le Morvan V. Robert J. (2012). Short curcumin treatment modulates oxidative stress, arginase activity, aberrant crypt foci, and TGF-beta 1 and HES-1 transcripts in 1,2-dimethylhydrazine-colon carcinogenesis in mice. Toxicology 302, 308–317. 10.1016/j.tox.2012.08.014 22982865

[B23] Bray F. Laversanne M. Sung H. Ferlay J. Siegel R. L. Soerjomataram I. (2024). Global cancer statistics 2022: GLOBOCAN estimates of incidence and mortality worldwide for 36 cancers in 185 countries. CA Cancer J. Clin. 74 (3), 229–263. 10.3322/caac.21834 38572751

[B24] Brent R. L. (2004). Utilization of animal studies to determine the effects and human risks of environmental toxicants (drugs, chemicals, and physical agents). Pediatrics 113 (4), 984–995. 10.1542/peds.113.s3.984 15060191

[B25] Bueno-Pereira T. O. Bertozzi-Matheus M. Zampieri G. M. Abbade J. F. Cavalli R. C. Nunes P. R. (2022). Markers of endothelial dysfunction Are attenuated by resveratrol in preeclampsia. Antioxidants 11, 2111. 10.3390/antiox11112111 36358483 PMC9686533

[B26] Bunch K. L. Abdelrahman A. A. Caldwell R. B. Caldwell R. W. (2022). Novel therapeutics for diabetic retinopathy and diabetic macular edema: a pathophysiologic perspective. Front. Physiol. 13, 831616. 10.3389/fphys.2022.831616 35250632 PMC8894892

[B27] Buscher K. Ehinger E. Gupta P. Pramod A. B. Wolf D. Tweet G. (2017). Natural variation of macrophage activation as disease-relevant phenotype predictive of inflammation and cancer survival. Nat. Commun. 8, 16041. 10.1038/ncomms16041 28737175 PMC5527282

[B28] Bygd H. C. Ma L. Bratlie K. M. (2017). Physicochemical properties of liposomal modifiers that shift macrophage phenotype. Mater. Sci. Eng. 79, 237–244. 10.1016/j.msec.2017.05.032 28629014

[B29] Caldwell R. B. Zhang W. B. Romero M. J. Caldwell R. W. (2010). Vascular dysfunction in retinopathy-An emerging role for arginase. Brain Res. Bull. 81 (2-3), 303–309. 10.1016/j.brainresbull.2009.08.025 19737603 PMC2815222

[B30] Canali M. M. Porporatto C. Aoki M. P. Bianco I. D. Correa S. G. (2010). Signals elicited at the intestinal epithelium upon chitosan feeding contribute to immunomodulatory activity and biocompatibility of the polysaccharide. Vaccine 28, 5718–5724. 10.1016/j.vaccine.2010.06.027 20598784

[B145] Carter N. S. Stamper B. D. Elbarbry F. Nguyen V. Lopez S. Kawasaki Y. (2021). Natural products that target the arginase in *Leishmania* parasites hold therapeutic promise. Microorganisms. 9 (2), 267. 10.3390/microorganisms9020267 PMC7911663 33525448

[B31] Cerkezkayabekir A. Sanal F. Bakar E. Ulucam E. Inan M. (2017). Naringin protects viscera from ischemia/reperfusion injury by regulating the nitric oxide level in a rat model. Biotech. Histochem. 92, 252–263. 10.1080/10520295.2017.1305499 28426254

[B32] Chauhan S. Nusbaum R. J. Huante M. B. Holloway A. J. Endsley M. A. Gelman B. B. (2024). Therapeutic modulation of Arginase with nor-NOHA alters immune responses in experimental mouse models of pulmonary tuberculosis including in the setting of human immunodeficiency virus (HIV) co-infection. Trop. Med. Infect. Dis. 9, 129. 10.3390/tropicalmed9060129 38922041 PMC11209148

[B33] Chen B. Xue J. Meng X. Slutzky J. L. Calvert A. E. Chicoine L. G. (2014). Resveratrol prevents hypoxia-induced arginase II expression and proliferation of human pulmonary artery smooth muscle cells via Akt-dependent signaling. Am. J. Physiol. Lung Cell. Mol. Physiol. 307, L317–L325. 10.1152/ajplung.00285.2013 24951775 PMC4137162

[B34] Chen C. L. Hsu S. C. Ann D. K. Yen Y. Kung H. J. (2021). Arginine signaling and cancer metabolism. Cancers 13, 3541. 10.3390/cancers13143541 34298755 PMC8306961

[B35] Cheng J. L. Long J. Zhang J. J. Han L. Hu Y. F. Liu J. H. (2023). Safety, tolerance, and pharmacokinetics of salvianolic acid B in healthy Chinese volunteers: a randomized, double-blind, placebo-controlled phase 1 clinical trial. Front. Pharmacol. 14, 1146309. 10.3389/fphar.2023.1146309 37124221 PMC10133543

[B36] Choi C. I. Koo B. H. Hong D. Kwon H. J. Hoe K. L. Won M. H. (2019). Resveratrol is an arginase inhibitor contributing to vascular smooth muscle cell vasoconstriction via increasing cytosolic calcium. Mol. Med. Rep. 19, 3767–3774. 10.3892/mmr.2019.10035 30896798

[B37] Chow H. H. S. Hakim I. A. (2011). Pharmacokinetic and chemoprevention studies on tea in humans. Pharmacol. Res. 64 (2), 105–112. 10.1016/j.phrs.2011.05.007 21624470 PMC3152306

[B38] Chudzinska M. Rogowicz D. Wolowiec L. Banach J. Sielski S. Bujak R. (2021). Resveratrol and cardiovascular system-the unfulfilled hopes. Ir. J. Med. Sci. 190, 981–986. 10.1007/s11845-020-02441-x 33219913

[B39] Crombez E. A. Cederbaum S. D. (2005). Hyperargininemia due to liver arginase deficiency. Mol. Genet. Metab. 84, 243–251. 10.1016/j.ymgme.2004.11.004 15694174

[B40] da Silva A. B. Cerqueira Coelho P. L. Oliveira M. d.N. Oliveira J. L. Oliveira Amparo J. A. da Silva K. C. (2020). The flavonoid rutin and its aglycone quercetin modulate the microglia inflammatory profile improving antiglioma activity. Brain, Behav. Immun. 85, 170–185. 10.1016/j.bbi.2019.05.003 31059805

[B41] da Silva E. R. Brogi S. Grillo A. Campiani G. Gemma S. Vieira P. C. (2019a). Cinnamic acids derived compounds with antileishmanial activity target Leishmania amazonensis arginase. Chem. Biol. Drug Des. 93, 139–146. 10.1111/cbdd.13391 30216691

[B42] da Silva E. R. Brogi S. Lucon-Junior J. F. Campiani G. Gemma S. Maquiaveli C. d.C. (2019b). Dietary polyphenols rutin, taxifolin and quercetin related compounds target Leishmania amazonensis arginase. Food Funct. 10, 3172–3180. 10.1039/c9fo00265k 31134235

[B43] Dedkova E. N. Blatter L. A. (2009). Characteristics and function of cardiac mitochondrial nitric oxide synthase. J. Physiol. (London, U. K.) 587, 851–872. 10.1113/jphysiol.2008.165423 PMC2669975 19103678

[B44] Ding Y. Liu P. Chen Z.-L. Zhang S.-J. Wang Y.-Q. Cai X. (2018). Emodin attenuates lipopolysaccharide-induced acute liver injury via inhibiting the TLR4 signaling pathway *in vitro* and *in vivo* . Front. Pharmacol. 9, 962. 10.3389/fphar.2018.00962 30186181 PMC6113398

[B45] Djaldetti M. (2024). Immunomodulatory and chemopreventive effects of resveratrol on the digestive system cancers. Oncol. Res. 32, 1389–1399. 10.32604/or.2024.049745 39220125 PMC11361903

[B46] Ellwood L. Torun G. Bahar Z. Fernandez R. (2019). Effects of flavonoid-rich fruits on hypertension in adults: a systematic review. JBI Database. Syst. Rev. Implement. Rep. 17, 2075–2105. 10.11124/jbisrir-d-19-00050 31464854

[B47] El-Sayed N. S. Khalil N. A. Saleh S. R. Aly R. G. Basta M. (2024). The possible neuroprotective effect of caffeic acid on cognitive changes and anxiety-like behavior occurring in young rats fed on high-fat diet and exposed to chronic stress: role of β-catenin/GSK-3B pathway. J. Mol. Neurosci. 74 (3), 61. 10.1007/s12031-024-02232-4 38954245

[B48] Eren R. Özaydin B. C. Göksoy E. Yigit Z. M. Özgür B. G. (2024). A case with autism spectrum disorder and concomitant arginase deficiency. J. Behcet. Uz. Child. Hosp. 14 (3), 195–198. 10.4274/jbuch.galenos.2024.39112

[B49] Erens C. Van Broeckhoven J. Bronckaers A. Lemmens S. Hendrix S. (2023). The dark side of an essential amino acid: L-Arginine in Spinal Cord Injury. J. Neurotrauma 40, 820–832. 10.1089/neu.2022.0271 36503258

[B50] Fan Y. Huang S. L. Li H. Cui Y. L. Li D. Y. (2021). Resveratrol attenuates inflammation by regulating macrophage polarization via inhibition of toll-like receptor 4/MyD88 signaling pathway. Pharmacogn. Mag. 17, 321–326. 10.4103/pm.pm_312_20

[B51] Gambardella J. Khondkar W. Morelli M. B. Wang X. J. Santulli G. Trimarco V. (2020). Arginine and endothelial function. Biomedicines 8, 277. 10.3390/biomedicines8080277 32781796 PMC7460461

[B52] Gao L. Zhang D. R. Chen M. H. Duan C. X. Dai W. T. Jia L. J. (2008). Studies on pharmacokinetics and tissue distribution of oridonin nanosuspensions. Int. J. Pharm. 355 (1-2), 321–327. 10.1016/j.ijpharm.2007.12.016 18242896

[B53] Garcia A. R. Oliveira D. M. P. Amaral A. C. F. Jesus J. B. Sodero A. C. R. Souza A. M. T. (2019). Leishmania infantum arginase: biochemical characterization and inhibition by naturally occurring phenolic substances. J. Enzyme Inhib. Med. Chem. 34 (1), 1100–1109. 10.1080/14756366.2019.1616182 31124384 PMC6534257

[B54] Girard-Thernier C. Pham T. N. Demougeot C. (2015). The promise of plant-derived substances as inhibitors of arginase. Mini-Rev. Med. Chem. 15, 798–808. 10.2174/1389557515666150511153852 25963565

[B55] Glanz V. Y. Orekhov A. N. Deykin A. V. (2018). Human disease modelling techniques: current progress. Curr. Mol. Med. 18 (10), 655–660. 10.2174/1566524019666190206204357 30727892

[B56] Golebiowski A. Beckett R. P. Van Zandt M. Ji M. K. Whitehouse D. Ryder T. R. (2013). 2-Substituted-2-amino-6-boronohexanoic acids as arginase inhibitors. Bioorg. Med. Chem. Lett. 23, 2027–2030. 10.1016/j.bmcl.2013.02.024 23453840

[B57] Goncalves dos Reis M. B. Manjolin L. C. Maquiaveli C. d.C. Santos-Filho O. A. da Silva E. R. (2013). Inhibition of leishmania (Leishmania) amazonensis and rat arginases by green tea EGCG, (+)-catechin and (2)-epicatechin: a comparative structural analysis of enzyme-inhibitor interactions. PLoS One 8, e78387. 10.1371/journal.pone.0078387 24260115 PMC3832641

[B58] Gong H. L. Tang W. F. Wang H. Xia Q. Huang X. (2011). Effects of food and gender on the pharmacokinetics of rhein and emodin in rats after oral dosing with Da-Cheng-Qi decoction. Phytother. Res. 25 (1), 74–80. 10.1002/ptr.3223 20623608

[B59] Graefe E. U. Wittig J. Mueller S. Riethling A. K. Uehleke B. Drewelow B. (2001). Pharmacokinetics and bioavailability of quercetin glycosides in humans. J. Clin. Pharmacol. 41 (5), 492–499. 10.1177/00912700122010366 11361045

[B60] Han H. W. Zhang Y. W. Peng G. D. Li L. W. Yang J. Yuan Y. (2021). Extracellular PKM2 facilitates organ-tissue fibrosis progression. Iscience 24 (10), 103165. 10.1016/j.isci.2021.103165 34693222 PMC8517170

[B61] Haworth S. M. M. Zhuge Z. B. Nihlén C. Von Rosen M. F. Weitzberg E. Lundberg J. O. (2021). Red blood cells from patients with pre-eclampsia induce endothelial dysfunction. J. Hypertens. 39, 1628–1641. 10.1097/hjh.0000000000002834 33657586

[B62] Hemmat N. Derakhshani A. Bannazadeh Baghi H. Silvestris N. Baradaran B. De Summa S. (2020). Neutrophils, crucial, or harmful immune cells involved in coronavirus infection: a bioinformatics study. Front. Genet. 11, 641. 10.3389/fgene.2020.00641 32582303 PMC7296827

[B63] Hua H. Y. Zhao Y. X. Liu L. Ye Q. X. Ge S. W. (2008). High-performance liquid chromatographic and pharmacokinetic analyses of an intravenous submicron emulsion of perillyl alcohol in rats. J. Pharm. Biomed. Anal. 48 (4), 1201–1205. 10.1016/j.jpba.2008.08.015 18849133

[B64] Huang C. Y. Wang J. Liu H. B. Huang R. Yan X. W. Song M. Y. (2022). Ketone body β-hydroxybutyrate ameliorates colitis by promoting M2 macrophage polarization through the STAT6-dependent signaling pathway. BMC Med. 20, 148. 10.1186/s12916-022-02352-x 35422042 PMC9011974

[B65] Islam T. Koboziev I. Albracht-Schulte K. Mistretta B. Scoggin S. Yosofvand M. (2021). Curcumin reduces adipose tissue inflammation and alters gut microbiota in diet-induced obese male mice. Mol. Nutr. Food Res. 65, e2100274. 10.1002/mnfr.202100274 34510720

[B66] Jackson J. Dietrich C. H. (2024). Synergistic antibacterial effects of gallate containing compounds with silver nanoparticles in gallate crossed linked PVA hydrogel films. Antibiot. (Basel) 13, 312. 10.3390/antibiotics13040312 PMC11047530 38666988

[B67] Joe Y. Zheng M. Kim H. J. Kim S. Uddin M. J. Park C. (2012). Salvianolic acid B exerts vasoprotective effects through the modulation of heme oxygenase-1 and arginase activities. J. Pharmacol. Exp. Ther. 341, 850–858. 10.1124/jpet.111.190736 22442118

[B68] Johnson F. K. Johnson R. A. Peyton K. J. Shebib A. R. Durante W. (2013). Arginase promotes skeletal muscle arteriolar endothelial dysfunction in diabetic rats. Front. Immunol. 4, 119. 10.3389/fimmu.2013.00119 23730303 PMC3657690

[B69] Johnson R. A. Durante W. Craig T. Peyton K. J. Myers J. G. Stewart R. M. (2010). Vascular arginase contributes to arteriolar endothelial dysfunction in a rat model of hemorrhagic shock. J. Trauma. 69 (2), 384–391. 10.1097/TA.0b013e3181e771a3 20699748

[B70] Kanaze F. I. Bounartzi M. I. Georgarakis M. Niopas I. (2007). Pharmacokinetics of the citrus flavanone aglycones hesperetin and naringenin after single oral administration in human subjects. Eur. J. Clin. Nutr. 61 (4), 472–477. 10.1038/sj.ejcn.1602543 17047689

[B71] Kar B. Rout S. R. Halder J. Mahanty R. Mishra A. Saha I. (2024). The recent development of luteolin-loaded nanocarrier in targeting cancer. Curr. Pharm. Des. 30, 2129–2141. 10.2174/0113816128313713240628063301 38963114

[B72] Kararli T. T. (1995). Comparison of the gastrointestinal anatomy, physiology, and biochemistry of humans and commonly used laboratory-animals. Biopharm. Drug Dispos. 16 (5), 351–380. 10.1002/bdd.2510160502 8527686

[B73] Kim A. Ma J. Y. (2019). Piceatannol-3-O-beta-D-glucopyranoside (PG) exhibits *in vitro* anti-metastatic and anti-angiogenic activities in HT1080 malignant fibrosarcoma cells. Phytomedicine 57, 95–104. 10.1016/j.phymed.2018.12.017 30668328

[B74] Kim N. N. Cox J. D. Baggio R. F. Emig F. A. Mistry S. K. Harper S. L. (2001). Probing erectile function: S-(2-boronoethyl)-L-cysteine binds to arginase as a transition state analogue and enhances smooth muscle relaxation in human penile corpus cavernosum. Biochemistry 40 (9), 2678–2688. 10.1021/bi002317h 11258879

[B75] Krause B. J. Del Rio R. Moya E. A. Marquez-Gutierrez M. Casanello P. Iturriaga R. (2015). Arginase-endothelial nitric oxide synthase imbalance contributes to endothelial dysfunction during chronic intermittent hypoxia. J. Hypertens. 33 (3), 515–524. 10.1097/hjh.0000000000000453 25629363

[B76] Kumari N. Bansal S. (2021). Arginine depriving enzymes: applications as emerging therapeutics in cancer treatment. Cancer Chemother. Pharmacol. 88, 565–594. 10.1007/s00280-021-04335-w 34309734

[B77] Lacchini R. Muniz J. J. Nobre Y. Cologna A. J. Martins A. C. P. Tanus-Santos J. E. (2015). Relationship between Arginase 1 and Arginase 2 levels and genetic polymorphisms with erectile dysfunction. Nitric Oxide 51, 36–42. 10.1016/j.niox.2015.10.003 26537638

[B78] Lan T. Dong Y. Shi J. Wang X. Xu Z. Zhang Y. (2024). Advancing self-healing soy protein hydrogel with dynamic Schiff base and metal-ligand bonds for diabetic chronic wound recovery. Aggregate 2024, e639. 10.1002/agt2.639

[B79] Landis-Piwowar K. Chen D. Foldes R. Chan T.-H. Dou Q. P. (2013). Novel epigallocatechin gallate analogs as potential anticancer agents: a patent review (2009-present). Expert Opin. Ther. Pat. 23, 189–202. 10.1517/13543776.2013.743993 23230990 PMC3840390

[B80] Lang G. P. Li C. Han Y. Y. (2021). Rutin pretreatment promotes microglial M1 to M2 phenotype polarization. Neural Regen. Res. 16 (12), 2499–2504. 10.4103/1673-5374.313050 33907040 PMC8374565

[B81] Lange P. S. Langley B. Lu P. Y. Ratan R. R. (2004). Novel roles for arginase in cell survival, regeneration, and translation in the central nervous system. J. Nutr. 134 (10), 2812S–2819S. 10.1093/jn/134.10.2812S 15465791

[B82] Li D. R. Zhang H. J. Lyons T. W. Lu M. Achab A. Pu Q. L. (2021). Comprehensive strategies to bicyclic prolines: applications in the synthesis of potent arginase inhibitors. ACS Med. Chem. Lett. 12 (11), 1678–1688. 10.1021/acsmedchemlett.1c00258 34795856 PMC8591728

[B83] Li H. Li L. Mei H. Pan G. Wang X. Huang X. (2020). Antitumor properties of triptolide: phenotype regulation of macrophage differentiation. Cancer Biol. Ther. 21, 178–188. 10.1080/15384047.2019.1679555 31663424 PMC7012063

[B84] Li L. Liu R. He J. Li J. Guo J. Chen Y. (2022a). Naringin regulates microglia BV-2 activation and inflammation via the JAK/STAT3 pathway. Evid. Based Complement. Altern. Med. 2022, 3492058. 10.1155/2022/3492058 PMC9135528 35646153

[B85] Li M. H. Gong J. Gao L. X. Zou T. Kang J. H. Xu H. W. (2022b). Advanced human developmental toxicity and teratogenicity assessment using human organoid models. Ecotoxicol. Environ. Saf. 235, 113429. 10.1016/j.ecoenv.2022.113429 35325609

[B86] Lin L. C. Pai Y. F. Tsai T. H. (2015). Isolation of luteolin and luteolin-7-O-glucoside from dendranthema morifolium ramat tzvel and their pharmacokinetics in rats. J. Agric. Food Chem. 63 (35), 7700–7706. 10.1021/jf505848z 25625345

[B87] Lin M. H. Cheng P. C. Hsiao P. J. Chen S. C. Hung C. H. Kuo C. H. (2023). The GLP-1 receptor agonist exenatide ameliorates neuroinflammation, locomotor activity, and anxiety-like behavior in mice with diet-induced obesity through the modulation of microglial M2 polarization and downregulation of SR-A4. Int. Immunopharmacol. 115, 109653. 10.1016/j.intimp.2022.109653 36587502

[B88] Lisi L. Pizzoferrato M. Miscioscia F. T. Topai A. Navarra P. (2017). Interactions between integrase inhibitors and human arginase 1. J. Neurochem. 142, 153–159. 10.1111/jnc.14039 28397245

[B89] Lisi L. Tramutola A. Navarra P. Dello Russo C. (2014). Antiretroviral agents increase NO production in gp120/IFNγ-stimulated cultures of rat microglia via an arginase-dependent mechanism. J. Neuroimmunol. 266, 24–32. 10.1016/j.jneuroim.2013.10.013 24268674

[B90] Liu D. You M. Xu Y. Li F. Zhang D. Li X. (2016). Inhibition of curcumin on myeloid-derived suppressor cells is requisite for controlling lung cancer. Int. Immunopharmacol. 39, 265–272. 10.1016/j.intimp.2016.07.035 27497194

[B91] Liu F. L. Yao Y. F. Guo C. X. Dai P. Y. Huang J. H. Peng P. (2024). Trichodelphinine A alleviates pulmonary fibrosis by inhibiting collagen synthesis via NOX4-ARG1/TGF-β signaling pathway. Phytomedicine 132, 155755. 10.1016/j.phymed.2024.155755 38870750

[B92] Lubinski D. Thompson T. (1993). Species and individual-differences in communication based on private states. Behav. Brain Sci. 16 (4), 627–642. 10.1017/s0140525x00032039

[B146] Manjolin L. C. dos Reis M. B. Maquiaveli Cdo C. Santos-Filho O. A. da Silva E. R. (2013). Dietary flavonoids fisetin, luteolin and their derived compounds inhibit arginase, a central enzyme in Leishmania (Leishmania) amazonensis infection. Food Chem. 141 (3), 2253–2262. 10.1016/j.foodchem.2013.05.025 23870955

[B93] McNutt M. C. Foreman N. Gotway G. (2023). Arginase 1 deficiency in patients initially diagnosed with hereditary spastic paraplegia. M. D. C. P. 10, 109–114. 10.1002/mdc3.13612 PMC9847303 36698992

[B94] Minozzo B. R. Fernandes D. Beltrame F. L. (2018). Phenolic compounds as arginase inhibitors: new insights regarding endothelial dysfunction treatment. Planta Med. 84, 277–295. 10.1055/s-0044-100398 29342480

[B95] Molaro M. C. Battisegola C. Schiano M. E. Failla M. Rimoli M. G. Lazzarato L. (2025). Synthesis of arginase inhibitors: an overview. Pharmaceutics 17 (1), 117. 10.3390/pharmaceutics17010117 39861764 PMC12068017

[B96] Moretto J. Girard C. Demougeot C. (2019). The role of arginase in aging: a systematic review. Exp. Gerontol. 116, 54–73. 10.1016/j.exger.2018.12.011 30578842

[B97] Morris S. M. (2002). Regulation of enzymes of the urea cycle and arginine metabolism. Annu. Rev. Nutr. 22, 87–105. 10.1146/annurev.nutr.22.110801.140547 12055339

[B98] Muller J. Attia R. Zedet A. Girard C. Pudlo M. (2022). An update on arginase inhibitors and inhibitory assays. Mini-Rev. Med. Chem. 22, 1963–1976. 10.2174/1389557522666211229105703 34967285

[B99] Niu F. L. Yu Y. Li Z. Z. Ren Y. Y. Li Z. Ye Q. (2022). Arginase: an emerging and promising therapeutic target for cancer treatment. Biomed. Pharmacother. 149, 112840. 10.1016/j.biopha.2022.112840 35316752

[B100] Oboh G. Ojueromi O. O. Ademosun A. O. Omayone T. P. Oyagbemi A. A. Ajibade T. O. (2021). Effects of caffeine and caffeic acid on selected biochemical parameters in L-NAME-induced hypertensive rats. J. Food Biochem. 45, e13384. 10.1111/jfbc.13384 32725646

[B101] Patra S. Pradhan B. Nayak R. Behera C. Rout L. Jena M. (2021). Chemotherapeutic efficacy of curcumin and resveratrol against cancer: chemoprevention, chemoprotection, drug synergism and clinical pharmacokinetics. Semin. Cancer Biol. 73, 310–320. 10.1016/j.semcancer.2020.10.010 33152486

[B102] Pavuluri H. Jose M. Fasaludeen A. Sundaram S. Radhakrishnan A. Banerjee M. (2023). Arginase deficiency-An unheralded cause of developmental epileptic encephalopathy. Epileptic Disord. 25, 556–561. 10.1002/epd2.20081 37243436

[B103] Peng X. McClements D. J. Liu X. Liu F. (2024). EGCG-based nanoparticles: synthesis, properties, and applications. Crit. Rev. Food Sci. Nutr. 22, 1–22. 10.1080/10408398.2024.2328184 38520117

[B104] Pérez S. Rius-Pérez S. (2022). Macrophage polarization and reprogramming in acute inflammation: a redox perspective. Antioxidants 11, 1394. 10.3390/antiox11071394 35883885 PMC9311967

[B105] Pham T. N. Bordage S. Pudlo M. Demougeot C. Thai K. M. Girard-Thernier C. (2016). Cinnamide derivatives as mammalian arginase inhibitors: synthesis, biological evaluation and molecular docking. Int. J. Mol. Sci. 17 (10), 1656. 10.3390/ijms17101656 27690022 PMC5085689

[B106] Pham T. N. Liagre B. Girard-Thernier C. Demougeot C. (2018). Research of novel anticancer agents targeting arginase inhibition. Drug Discov. Today 23 (4), 871–878. 10.1016/j.drudis.2018.01.046 29391126

[B107] Pudlo M. Demougeot C. Girard-Thernier C. (2017). Arginase inhibitors: a rational approach over one century. Med. Res. Rev. 37, 475–513. 10.1002/med.21419 27862081

[B108] Rath M. Müller I. Kropf P. Closs E. I. Munder M. (2014). Metabolism via arginase or nitric oxide synthase: two competing arginine pathways in macrophages. Front. Immunol. 5, 532. 10.3389/fimmu.2014.00532 25386178 PMC4209874

[B109] Sanoh S. Ohta S. (2014). Chimeric mice transplanted with human hepatocytes as a model for prediction of human drug metabolism and pharmacokinetics. Biopharm. Drug Dispos. 35 (2), 71–86. 10.1002/bdd.1864 24114658

[B110] Scaglia F. Lee B. (2006). Clinical, biochemical, and molecular spectrum of hyperargininemia due to arginase I deficiency. Am. J. Med. Genet. Part C 142C (2), 113–120. 10.1002/ajmg.c.30091 16602094 PMC4052756

[B111] Schlune A. vom Dahl S. Haeussinger D. Ensenauer R. Mayatepek E. (2015). Hyperargininemia due to arginase I deficiency: the original patients and their natural history, and a review of the literature. Amino Acids 47, 1751–1762. 10.1007/s00726-015-2032-z 26123990

[B112] Semis H. S. Kandemir F. M. Caglayan C. Kaynar O. Genc A. Arikan S. M. (2022). Protective effect of naringin against oxaliplatin-induced peripheral neuropathy in rats: a behavioral and molecular study. J. Biochem. Mol. Toxicol. 36, e23121. 10.1002/jbt.23121 35670529

[B113] Sharma S. Ali A. Ali J. Sahni J. K. Baboota S. (2013). Rutin: therapeutic potential and recent advances in drug delivery. Expert Opin. Invest. Drugs 22, 1063–1079. 10.1517/13543784.2013.805744 23795677

[B114] Shin H. Cama E. Christianson D. W. (2004). Design of amino acid aldehydes as transition-state analogue inhibitors of arginase. J. Am. Chem. Soc. 126, 10278–10284. 10.1021/ja047788w 15315440

[B115] Siavelis J. C. Bourdakou M. M. Athanasiadis E. I. Spyrou G. M. Nikita K. S. (2016). Bioinformatics methods in drug repurposing for Alzheimer's disease. Briefings Bioinf 17, 322–335. 10.1093/bib/bbv048 26197808

[B116] Sin Y. Y. Baron G. Schulze A. Funk C. D. (2015). Arginase-1 deficiency. J. Mol. Med. 93, 1287–1296. 10.1007/s00109-015-1354-3 26467175

[B117] Song Y.-d. Li X.-z. Wu Y.-x. Shen Y. Liu F.-f. Gao P.-p. (2018). Emodin alleviates alternatively activated macrophage and asthmatic airway inflammation in a murine asthma model. Acta Pharmacol. Sin. 39, 1317–1325. 10.1038/aps.2017.147 29417945 PMC6289379

[B118] Tariq H. Mukhtar S. Naz S. (2017). A novel mutation in ALS2 associated with severe and progressive infantile onset of spastic paralysis. J. Neurogenet. 31, 26–29. 10.1080/01677063.2017.1324441 28502191

[B119] Therrell B. L. Currier R. Lapidus D. Grimm M. Cederbaum S. D. (2017). Newborn screening for hyperargininemia due to arginase 1 deficiency. Mol. Genet. Metab. 121, 308–313. 10.1016/j.ymgme.2017.06.003 28659245

[B120] Tratsiakovich Y. Yang J. N. Gonon A. T. Sjöquist P. O. Pernow J. (2013). Arginase as a target for treatment of myocardial ischemia-reperfusion injury. Eur. J. Pharmacol. 720, 121–123. 10.1016/j.ejphar.2013.10.040 24183975

[B121] Trieu Dieu Linh N. Ryoo S. Choi H. (2016). Effect of resveratrol as an arginase inhibitor on vascular smooth muscle cell proliferation induced by native low-density lipoprotein. J. Hypertens. 34, 285–286. 10.1097/01.hjh.0000500673.55427.7e

[B122] Trujillo J. Chirino Y. I. Molina-Jijón E. Andérica-Romero A. C. Tapia E. Pedraza-Chaverrí J. (2013). Renoprotective effect of the antioxidant curcumin: recent findings. Redox Biol. 1, 448–456. 10.1016/j.redox.2013.09.003 24191240 PMC3814973

[B123] Vahter M. Gochfeld M. Casati B. Thiruchelvam M. Falk-Filippson A. Kavlock R. (2007). Implications of gender differences for human health risk assessment and toxicology. Environ. Res. 104 (1), 70–84. 10.1016/j.envres.2006.10.001 17098226

[B124] Veglia F. Sanseviero E. Gabrilovich D. I. (2021). Myeloid-derived suppressor cells in the era of increasing myeloid cell diversity. Nat. Rev. Immunol. 21, 485–498. 10.1038/s41577-020-00490-y 33526920 PMC7849958

[B125] Wang D. Li S. P. Fu J. S. Zhang S. Bai L. Guo L. (2016). Resveratrol defends blood-brain barrier integrity in experimental autoimmune encephalomyelitis mice. J. Neurophysiol. 116, 2173–2179. 10.1152/jn.00510.2016 27535376 PMC5102308

[B126] Wang D. D. He C.-Y. Wu Y.-J. Xu L. Shi C. Olatunji O. J. (2022). AMPK/SIRT1 deficiency drives adjuvant-induced arthritis in rats by promoting glycolysis-mediated monocytes inflammatory polarization. J. Inflamm. Res. 15, 4663–4675. 10.2147/jir.S378090 35996683 PMC9392262

[B127] Wang Y. Lu M. Tang D. (2018). Novel photoluminescence enzyme immunoassay based on supramolecular host-guest recognition using L-arginine/6-aza-2-thiothymine-stabilized gold nanocluster. Biosens. Bioelectron. 109, 70–74. 10.1016/j.bios.2018.03.007 29529510

[B128] Wiechert P. Marescau B. De Deyn P. P. Lowenthal A. (1989). Hyperargininemia, epilepsy and the metabolism of guanidino compounds. Padiatr Grenzgeb. 28 (2), 101–106. 2657590

[B129] World Health Organization (2023). Hypertension. Available online at: https://www.who.int/zh/news-room/fact-sheets/detail/hypertension (Accessed March 16, 2023).

[B130] Wu G. Y. Morris S. M. (1998). Arginine metabolism: nitric oxide and beyond. Biochem. J. 336, 1–17. 10.1042/bj3360001 9806879 PMC1219836

[B131] Wu W. Jiang X. Zeng Q. Zou H. Deng C. (2024). Facile and green synthesis of Au nanoparticles decorated Epigallocatechin-3-Gallate nanospheres with enhanced performance in stability, photothermal conversion and nanozymatic activity. Biomater. Adv. 166, 214050. 10.1016/j.bioadv.2024.214050 39317045

[B132] Wu Y. Xu Y. Yang A. Shen S. Cao Y. (2020). Comparative *in vivo* pharmacokinetics study of affeic acid, isoferulic acid and ferulic acid in crude and three different prepared Cimicifuga foetida L. Biomed. Chromatogr. 34 (9), e4868. 10.1002/bmc.4868 32335934

[B133] Wu Y. Z. Yi M. Niu M. K. Mei Q. Wu K. M. (2022). Myeloid-derived suppressor cells: an emerging target for anticancer immunotherapy. Mol. Cancer 21, 184. 10.1186/s12943-022-01657-y 36163047 PMC9513992

[B134] Xu P. Yan F. Zhao Y. Chen X. Sun S. Wang Y. (2020). Green tea polyphenol EGCG attenuates MDSCs-mediated immunosuppression through canonical and non-canonical pathways in a 4T1 murine breast cancer model. Nutrients 12, 1042. 10.3390/nu12041042 32290071 PMC7230934

[B135] Xu Y. Zhang Y. F. Chen X. Y. Zhong D. F. (2018). CYP3A4 inducer and inhibitor strongly affect the pharmacokinetics of triptolide and its derivative in rats. Acta Pharmacol. Sin. 39 (8), 1386–1392. 10.1038/aps.2017.170 29283173 PMC6289341

[B136] Yacout G. A. Ghareeb D. A. El-Hamshary S. A. El-Sadek M. M. (2014). Biological effect of di (p-methylbenzoyl) diselenide (*in-vitro*) and its acute hepatotoxicity on rats (*in-vivo*). Iran. J. Pharm. Res. 13 (3), 893–898. 25276189 PMC4177649

[B137] Yang L. M. Chu Z. L. Liu M. Zou Q. Li J. Y. Liu Q. (2023). Amino acid metabolism in immune cells: essential regulators of the effector functions, and promising opportunities to enhance cancer immunotherapy. J. Hematol. Oncol. 16, 59. 10.1186/s13045-023-01453-1 37277776 PMC10240810

[B138] Ye P. H. Li C. Y. Cheng H. Y. Anuraga G. Wang C. Y. Chen F. W. (2023). A novel combination therapy of arginine deiminase and an arginase inhibitor targeting arginine metabolism in the tumor and immune microenvironment. Am. J. Cancer Res. 13 (5), 1952–1969. 37293150 PMC10244097

[B139] Zanier E. R. Pischiutta F. Riganti L. Marchesi F. Turola E. Fumagalli S. (2014). Bone marrow mesenchymal stromal cells drive protective M2 microglia polarization after brain trauma. Neurotherapeutics 11, 679–695. 10.1007/s13311-014-0277-y 24965140 PMC4121458

[B140] Zhang J. Zhou Y. Sun Y. Yan H. Han W. Wang X. (2019). Beneficial effects of Oridonin on myocardial ischemia/reperfusion injury: insight gained by metabolomic approaches. Eur. J. Pharmacol. 861, 172587. 10.1016/j.ejphar.2019.172587 31377155

[B141] Zhang M. Li Y. Han C. Chu S. Yu P. Cheng W. (2024). Biosynthesis of nanoparticles with green tea for inhibition of β-amyloid fibrillation coupled with ligands analysis. Int. J. Nanomed. 19, 4299–4317. 10.2147/ijn.S451070 PMC11102095 38766654

[B142] Zhang M. F. Wu Z. M. Salas S. S. Aguilar M. M. Trillos-Almanza M. C. Buist-Homan M. (2023). Arginase 1 expression is increased during hepatic stellate cell activation and facilitates collagen synthesis. J. Cell. Biochem. 124 (6), 808–817. 10.1002/jcb.30403 37042199

[B147] Zhao Z. Huang H. Ke S. Deng B. Wang YX Xu N. (2024). Triptolide inhibits the proinflammatory potential of myeloid-derived suppressor cells via reducing Arginase-1 in rheumatoid arthritis. Int. Immunopharmacol. 127, 111345. 10.1016/j.intimp.2023.111345 38086266

[B143] Zhou J. J. Ye W. Chen L. Li J. H. Zhou Y. J. Bai C. F. (2024). Triptolide alleviates cerebral ischemia/reperfusion injury via regulating the Fractalkine/CX3CR1 signaling pathway. Brain Res. Bull. 211, 110939. 10.1016/j.brainresbull.2024.110939 38574865

[B144] Zimmerman B. Kundu P. Rooney W. D. Raber J. (2021). The effect of high fat diet on cerebrovascular health and pathology: a species comparative review. Molecules 26 (11), 3406. 10.3390/molecules26113406 34199898 PMC8200075

